# Metastasis from the tumor interior and necrotic core formation are regulated by breast cancer-derived angiopoietin-like 7

**DOI:** 10.1073/pnas.2214888120

**Published:** 2023-02-28

**Authors:** Ami Yamamoto, Yin Huang, Brad A. Krajina, Margaux McBirney, Andrea E. Doak, Sixuan Qu, Carolyn L. Wang, Michael C. Haffner, Kevin J. Cheung

**Affiliations:** ^a^Translational Research Program, Public Health Sciences Division, Fred Hutchinson Cancer Center, Seattle, WA 98109; ^b^Human Biology Division, Fred Hutchinson Cancer Center, Seattle, WA 98109; ^c^Molecular and Cellular Biology Graduate Program, University of Washington, Seattle, WA 98195; ^d^Department of Radiology, University of Washington School of Medicine, Seattle, WA 98195; ^e^Division of Human Biology, Fred Hutchinson Cancer Center, Seattle, WA 98109; ^f^Division of Clinical Research, Fred Hutchinson Cancer Center, Seattle, WA 98109

**Keywords:** necrotic core, angiopoietin-like 7, circulating tumor cells, metastasis, breast cancer

## Abstract

Necrosis in the tumor interior is a common feature of aggressive cancers that is associated with poor clinical prognosis and the development of metastasis. How the necrotic core promotes metastasis remains unclear. Here, we report that emergence of necrosis inside the tumor is correlated temporally with increased tumor dissemination in a rat breast cancer model and in human breast cancer patients. By performing spatially focused transcriptional profiling, we identified angiopoietin-like 7 (Angptl7) as a tumor-specific factor localized to the perinecrotic zone. Functional studies showed that Angptl7 loss normalizes central necrosis, perinecrotic dilated vessels, metastasis, and reduces circulating tumor cell counts to nearly zero. Mechanistically, Angptl7 promotes vascular permeability and supports vascular remodeling in the perinecrotic zone. Taken together, these findings show that breast tumors actively produce factors controlling central necrosis formation and metastatic dissemination from the tumor core.

## Significance

Aggressive tumors often die from the inside out, a process called necrotic cell death. Necrosis is associated with dissemination of cancer cells but how necrosis promotes tumor dissemination is not understood. Here, we used rats as a model organism to increase detection of dissemination events. This uncovered a sharp rise in circulating tumor cell (CTC) abundance associated with necrosis and changes in the blood vessels. Gene expression analysis revealed a tumor-derived gene program involved in shaping the tumor core ecosystem. We demonstrate that necrosis, vascular remodeling, and metastatic dissemination are dependent on a tumor-secreted factor, angiopoietin-like 7 (Angptl7). Understanding the molecular factors regulating metastatic dissemination from the necrotic core could unveil therapeutic strategies to treat and prevent metastatic cancers.

The development of metastasis is the pivotal determinant of long-term survival in most human cancers (1). A key step in this process is the dissemination of tumor cells into the systemic circulation (2, 3). A long-standing question is where tumor cells disseminate from. A major focal point of investigation is the tumor-stromal border where cancer cells are directly observed to invade singly or collectively into surrounding tissues and disseminate into local blood vessels (4–7). Aiding this process are local conditions in the tumor microenvironment, such as hypoxia, acidity, and nutrient deficiency, in partnership with immune cells, that promote tumor dissemination (8–11). Counterintuitively, many of these microenvironmental influences are most prevalent in the tumor core, in regions of disordered tissue and blood vessel microarchitecture, where nutrient and oxygen availability are most limited (12, 13). One way to reconcile these competing observations is that tumor dissemination can also occur in the tumor core. Consistent with this hypothesis, the tumor core harbors abnormal biomechanics, increased interstitial pressure, and vascular leakiness thought conducive to dissemination (13–15). Further, tumor cells in the interior are actively migratory and spatially coordinated (16) and tumor cell intravasation was determined to occur almost exclusively in the tumor core in an avian model system (17). These observations suggest mechanisms for metastatic dissemination from the tumor core. However, owing to the spatial heterogeneity and cellular complexity of the tumor core ecosystem, the molecular factors regulating dissemination from within and the role of tumor cells, as active drivers or passive participants in this process, are not understood.

In the setting of extreme nutrient limitation and vascular compromise, tumor cells undergo necrotic cell death. Necrosis is a pervasive feature of many aggressive fast-growing tumors, associated with poor prognosis and markedly increased risk of metastasis (18–22). A challenge of between-tumor/between-patient association studies is that it is not possible to disentangle if necrosis is a regulator of metastasis or a by-product of other more aggressive features such as tumor grade or tumor subtype (23, 24). In this regard, spatial analyses within regions of the same tumor are suggestive. Spatial analyses of blood vessel invasion in lung cancers, sarcomas, hepatocellular carcinoma, and breast cancers have observed that between 25 to 50% of all blood vessel invasion events occur intratumorally in the tumor core (25–28). Likewise, spatial and temporal multiregional sequencing of primary and metastatic renal cell carcinomas reveal that metastatic subclones preferentially originate from the tumor interior where necrosis and increased copy number alterations predominate (29). Tumor cells that are dead cannot themselves give rise to metastasis. However, these clinical observations indicate that tumor cells neighboring the most intense regions of necrosis make productive contributions to metastatic dissemination. How the necrotic core promotes metastatic dissemination remains unclear.

In this study, we leveraged a rat transplantation model to uncover a temporal correlation between tumor dissemination and the formation of large contiguous zones of necrosis within the tumor core. These necrotic zones harbored dilated blood vessels and intravascular tumor cells. Importantly, our studies revealed a tumor-cell secreted factor angiopoietin like-7 (Angptl7) that regulates formation of necrosis. Our studies define a targetable axis of the necrosis ecosystem to suppress metastatic dissemination from the tumor interior.

## Results

### A Low-to-High Circulating Tumor Cell (CTC) Transition Occurs in a Rat Transplantation Model of Breast Cancer.

Isolating tumor cells in transit is painstaking in foundational models for cancer metastasis research including mouse, zebrafish, and chick embryo (17, 30–35). We hypothesized the use of rats, a larger animal model, would enable more robust detection of dissemination events locally and systemically in the circulation. In this study, we employed SCID rats on the Sprague–Dawley background with a double knockout for the Rag2 and Il2rgamma genes [denoted SRG (36)]. SRG rats are deficient in T cells, B cells, and NK cells similar to NOD scid gamma (NSG) mice. To compare the performance of these two transplantation models, we transplanted 4T1 tumor cells, a mouse mammary tumor model of triple-negative breast cancer, transduced with a GFP reporter, into the mammary fat pad of SRG rats or NSG mice and collected all tumors synchronously once maximum tumor size was reached for mice, which was 2 cm in our animal facility (*SI Appendix*, Fig. S1*A*). Altogether, SRG rats produced three times larger tumors by weight and by estimated tumor volume (*SI Appendix*, Fig. S1 *B* and *C*). Ten times more blood was collected with SRG rats, which in turn yielded 10 times more single CTCs and CTC-clusters per animal (*SI Appendix*, Fig. S1 *D*–*G*). For both CTCs and CTC-clusters, morphologic appearance and relative proportions in blood were identical between SRG rats and NSG mice (*SI Appendix*, Fig. S1 *E* and *H*). Consistent with the increased CTC abundance, lung metastases counted by stereomicroscopy were four times more abundant in SRG rats (*SI Appendix*, Fig. S1 *I *and *J*). Because the same number of 4T1 cells was transplanted in both SRG rats and NSG mice and because tumors were harvested at the same time, our direct comparison showed that the rat transplantation model increases the efficiency of detecting rare tumor dissemination events by 10-fold.

Having identified the rat xenograft model as an efficient system to study CTC dissemination, we next applied this model to determine CTC abundance temporally.

**Fig. 1.**
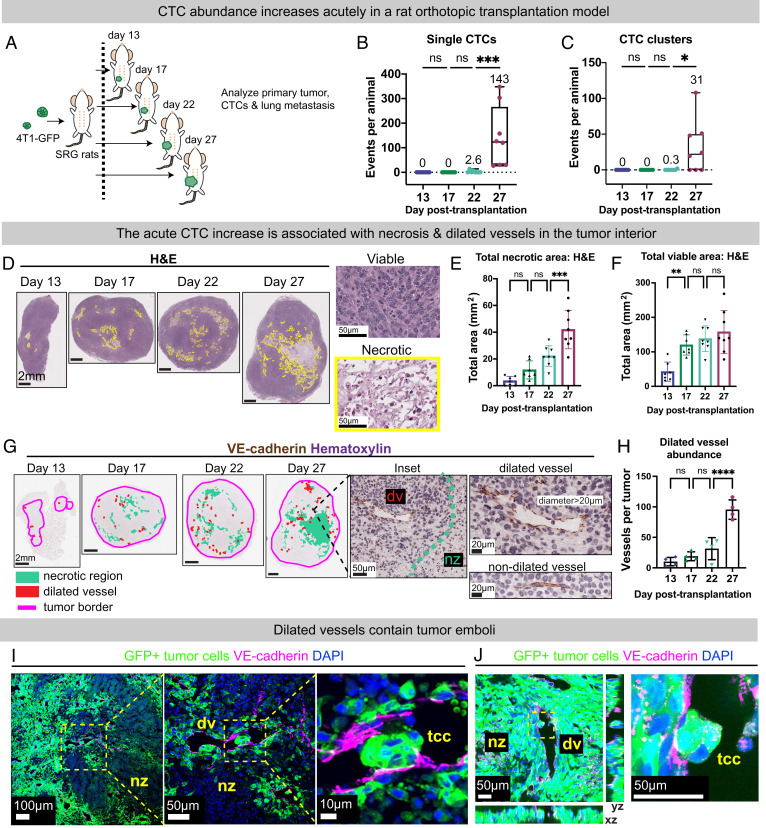
CTC transition is temporally associated with tumor necrosis, dilated vessels, and perinecrotic intravascular emboli. (*A*) Experimental schema. 4T1 mouse mammary tumor cells labeled with cytoplasmic GFP (4T1-GFP) were orthotopically transplanted into a single #4 mammary fat pad of SRG rats. Blood and tissues were harvested at day 13 (n = 7), 17 (n = 7), 22 (n = 8), and 27 (n = 8) posttransplantation. (*B* and *C*) Single CTC and CTC clusters per animal. Box plots shown with mean values labeled. (*D*) Representative hematoxylin and eosin (H&E) stains of day 13 to 27 rat tumors. (*Left*) Necrotic region indicated with yellow border. (*Right, * *Insets*) of necrotic and nonnecrotic regions from a day 27 tumor. (*E* and *F*) Total necrotic area and total viable area were determined by H&E. Mean ± SD. (*G*) Representative day 13 to day 27 rat tumors stained for VE-cadherin (DAB) and counterstained with hematoxylin. (*Left*) low power views. Necrotic regions shown in turquoise, dilated vessels in red, and tumor border in magenta. (*Right*) example of a dilated and nondilated vessel. Dv = dilated vessel. Nz = necrotic zone. (*H*) Number of VE-cadherin+ dilated blood vessels. Mean ± SD. (*I* and *J*) Representative immunofluorescent images of GFP+ tumor emboli inside VE-cadherin+ blood vessel from thin (10 µm) and thick (30 µm) tumor sections. (*I, Left*) low power image of perinecrotic zone. Insets showing high magnification of tumor cell cluster (tcc) within a dilated vessel (dv) (I, Middle and Right). (*J*) Confocal images from z-stack showing tumor cell cluster (tcc) within dilated vessel (dv). Xy, xz, and yz cross-sections shown. Magenta: VE-cadherin; Red: 594 conjugated lectin; Blue: DAPI; Green: tumor cells expressing GFP (membrane GFP in *I* & cytoplasmic GFP in *J*. nz = necrosis. *P*-values for *B*, *C*, *E*, *F*, and *H* determined by one-way ANOVA.

We transplanted GFP-labeled 4T1 cells into the mammary fat pad of SRG rats (Fig. 1*A*). Blood, lungs, and tumors were harvested at days 13, 17, 22, and 27 post-transplantation. In this model, tumor size increased significantly between days 13 and 17 with evidence of growth plateau and significant variance in tumor size between SRG rats by both estimated tumor volume and by tumor weight (*SI Appendix*, Fig. S2 *A* and *B*). Surprisingly in the same time frame, we observed a pronounced nonlinear increase in CTC abundance. Specifically, between days 22 and 27, we identified a ~50-fold increase in CTCs and ~10-fold increase in CTC clusters (Fig. 1 *B* and *C* and *SI Appendix*, Fig. S2 *C* and *D*).

### The Low-to-High CTC Transition Is Associated with a Necrotic Tumor Core, Dilated Vessels, and Perinecrotic Tumor Cell Vascular Invasion.

Given the robust increase in CTC abundance that was uncorrelated with primary tumor burden, we next asked how primary tumors were changing on a cellular level. Hematoxylin and eosin (H&E) staining of tumors revealed tumors that were histologically indistinguishable at the tumor-invasive border over time (*SI Appendix*, Fig. S2*E*). Instead, H&E revealed robust changes in the tumor core with appearance of confluent zones of central necrosis. Total necrotic area in the primary tumor increased over time with marked elevation between days 22 and 27, whereas total viable area increased early and subsequently plateaued (Fig. 1 *D*–*F*). As a second method, tetrazolium chloride assay, a colorimetric method for determining cell viability, confirmed that total necrotic area, but not total viable area, increased markedly at the late time point (*SI Appendix*, Fig. S2 *F*–*H*). Likewise, the number of lung metastases per animal was highly correlated with necrotic area, but not tumor volume (*SI Appendix*, Fig. S2 *I*–*K*).

Extending these results further, we stained tumors for VE-cadherin to mark blood vessels. VE-cadherin staining demonstrated abnormal dilated blood vessels, penetrating the necrotic core, that was markedly increased in abundance and density between days 22 and 27 (Fig. 1 *G* and *H* and  *SI Appendix*, Fig. S3 *A* and *B*). In contrast, neither total microvascular abundance nor density increased over the same time points (*SI Appendix*, Fig. S3 *C* and *D*). The number of lung metastases per animal and CTCs per animal were highly correlated with the number of dilated vessels but not total vessels (*SI Appendix*, Fig. S3 *E*–*H*). Functionally, dilated vessels were less often labeled by fluorescent lectin compared with nondilated vessels, indicative of differences in blood flow between vessel types (*SI Appendix*, Fig. S3 *I* and *J*). Further, dilated vessels contained GFP+ multicellular tumor cell clusters, consistent with vascular invasion (Fig. 1 *I* and *J*). Together, these data reveal an acute low-to-high CTC transition that coincides with robust changes in the tumor core, not rim. These tumor core alterations included confluent zones of necrosis and necrosis-adjacent dilated blood vessels containing intravascular tumor cells.

### Transcriptome Profiling Comparing Tumor Core and Rim Reveal That Angptl7 Is a Tumor-Specific, Core-Enriched Factor Localized to the Perinecrotic Zone.

**Fig. 2.**
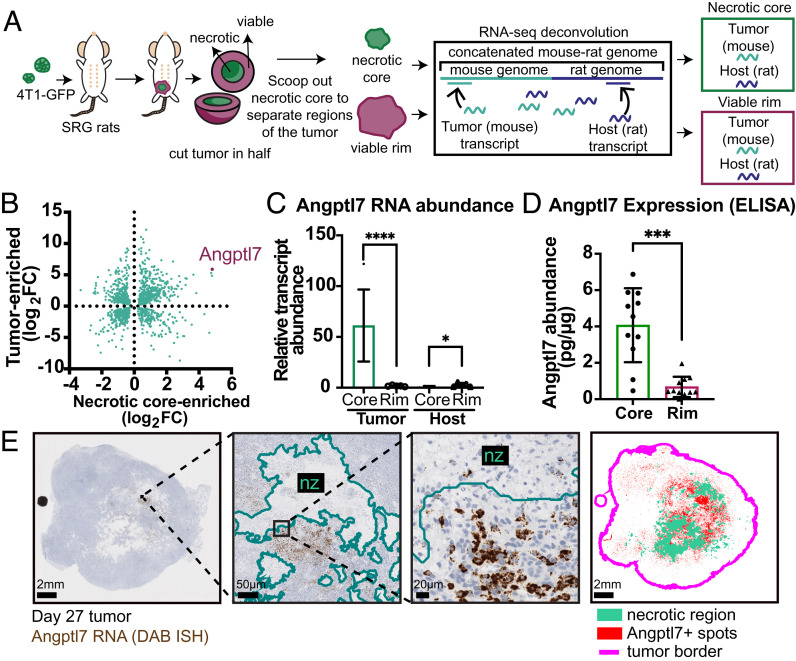
*Angptl7* is a tumor-derived, necrotic core-enriched transcript localized to the perinecrotic region of breast tumor. (*A*) Experimental schema. 4T1-GFP tumor cells were orthotopically transplanted into a single fat pad of SRG rats and collected between day 27 and day 30 (n = 5 animals). The harvested tumors were cut in half, and macrodissected to separate the necrotic core region from the nonnecrotic rim of the primary tumors. RNA was extracted and subjected to next-generation sequencing. Sequences were aligned to concatenated rat-mouse combined genome, and mouse and rat genes were deconvoluted from each other. (*B*) Mouse orthologs were identified for all rat genes and the relative RNA abundance per gene between tumor and host compartments was determined. The plot shows all genes with both differential enrichment for core:rim and for tumor:host with FDR ≤ 0.01. (*C*) Relative RNA abundance of *Angptl7* based on RNA-seq. Average expression and q-value (FDR) from multiple comparisons indicated on the graph. (*D*) ELISA quantification of Angptl7 protein abundance of the necrotic core and nonnecrotic rim regions of 4T1 tumors. Student *t* test. Mean ± SD. (*E*) Representative image of Angptl7 RNA in situ hybridization (ISH) of day 27 4T1 tumors. (*Left*) raw Angptl7 RNA ISH (brown). (*Right*) Detection by QuPath. Red: detection of Angptl7+ cells, turquoise: necrotic region, pink: tumor border. *P <0.05, **P < 0.01, ***P < 0.001, and ****P < 0.0001.

We next sought to define the molecular changes defining the necrotic core. To this end, we dissected out tumor core from the rim, and interrogated gene expression originating from tumor or from host cells (Fig. 2*A* and *SI Appendix*, Table S1). In this regard, the properties of the rat transplant model and the use of a mouse-in-rat xenograft were advantageous. At time of harvest, rat tumors were 3 cm in diameter or larger and had distinct tissue morphology between the firmer tumor rim and softer, pinker tumor core, that was in some cases also obviously liquified (*SI Appendix*, Fig. S4*A*), enabling us to reliably macrodissect the necrotic core from the nonnecrotic rim. Following RNA-sequencing of macrodissected regions, sequence reads were further aligned to a concatenated rat–mouse combined genome, and deconvoluted to obtain tumor-derived gene expression (originating from mouse) vs. host-derived gene expression (originating from the rat). This deconvolution algorithm robustly separated mouse and rat RNA transcriptomes and revealed that 76% of reads were mouse-in-origin in tumor rim, and 84% of reads were mouse-in-origin in tumor core (*SI Appendix*, Fig. S4*B*), indicating the majority of reads were tumor-derived.

Gene-ontology analysis further revealed distinct patterns of ontology enrichment between tumor and host, and between rim and core (*SI Appendix*, Fig. S4*C* and Table S2). In the host transcriptome, gene sets typically associated with the invasive border were up in the rim (including extracellular matrix, vascular morphogenesis, and locomotion), while gene sets associated with tissue necrosis were up in the core (including cellular stress response, RNA metabolism, and neutrophil degranulation). Likewise, in the tumor transcriptome, gene sets associated with replicating cells were up in the rim (cell cycle, DNA replication), while gene sets involved in vascular remodeling appeared up in the core (vasculature development/morphogenesis, matrisome, and regulation of cell adhesion).

These gene expression patterns revealed that tumor cells in the interior actively express genes associated with vascular tube morphogenesis. However, these core genes could be expressed in both tumor and host compartments. To illustrate, the top differentially expressed tumor-derived gene was *Camk1d.* However, host-derived *Camk1d* was also expressed in both core and rim; therefore, *Camk1d* is not tumor-specific (*SI Appendix*, Fig. S4*D*). To identify top core-enriched tumor-specific genes, we performed interspecies analysis to find genes that were specifically mouse tumor-derived and not expressed in the rat host (Fig. 2*B*). The top enriched factor from this analysis was angiopoietin-like 7 (*Angptl7*), enriched 32-fold in tumor vs. host, and over 16-fold in tumor core vs. tumor rim (Fig. 2*C*). The enrichment for Angptl7 in the tumor core was further confirmed by qPCR, western blot, and ELISA specific for mouse Angptl7 (Fig. 2*D* and *SI Appendix*, Fig S4 *E*–*G*). In addition, we performed RNA in situ hybridization (ISH) for mouse *Angptl7* (Fig. 2*E* and *SI Appendix*, Fig. S4*H*). To quantify spatial enrichment, we calculated the distance of every spot relative to the nearest tumor-stromal border or tumor-necrotic interface. *Angptl7* was markedly enriched in a perinecrotic distribution in the tumor core and with little expression in the tumor rim. These findings show that Angptl7 is a tumor-derived factor enriched in the core of high-CTC tumors, and spatially localized to the perinecrotic zone.

### Angptl7 Suppression Markedly Normalizes Histologic Necrosis in the Tumor Core and Reduces the Number of CTCs and Metastases.

**Fig. 3.**
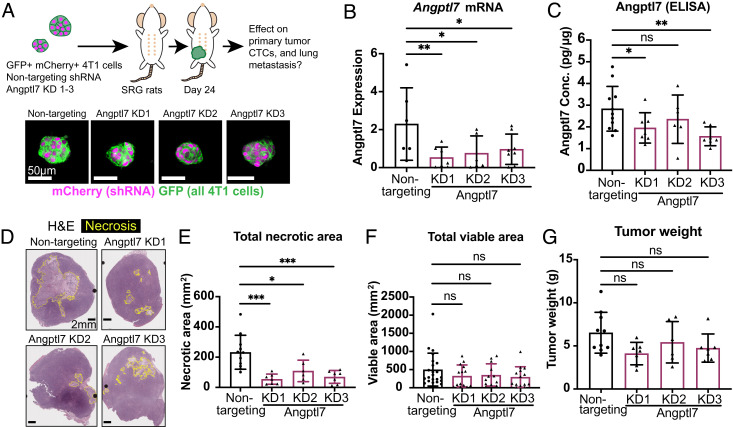
Suppression of Angptl7 normalizes tumor necrosis. (*A*) Experimental schema. 4T1 tumor cells labeled with membrane GFP and transduced with Angplt7 shRNA or nontargeting control were orthotopically transplanted into a single mammary fat pad of SRG rats. shRNA contained an mCherry tag, so cells expressing shRNA have cytoplasmic mCherry label. Blood, tumor, and lungs were collected. Nontargeting control (n = 11), Angptl7 knockdown KD1 (n = 7), Angptl7 KD2 (n = 6), Angptl7 KD3 (n = 7). (*B*) In vivo knockdown confirmation by qPCR based on tumor core. (*C*) ELISA confirmation by in vivo knockdown. Lysates were made from tumor necrotic core from Angptl7 knockdown and nontargeting control tumor cell transplantation experiments. (*D*) Representative hematoxylin and eosin (H&E) staining of primary tumors for Angptl7 knock down tumors and nontargeting control. Yellow borders indicate the necrotic regions. (*E* and *F*) Total necrotic area and total viable area based on H&E staining of Angptl7 KD and nontargeting control tumor. (*G*) Tumor weight of Angptl7 KD and nontargeting control tumor. All graphs shown display mean ± SD. Mean values shown on graphs. *P*-values for *B* and *C* determined by Mann–Whitney test; for *E*–*G* determined by ANOVA. *P <0.05, **P < 0.01, ***P < 0.001, and ****P < 0.0001.

ANGPTL7 belongs to a family of secreted proteins structurally related to the angiopoietins and that have been implicated in lipid metabolism, cardiovascular disease, stem cell renewal, and cancer (37–44). Compared with other ANGPTL proteins, ANGPTL7 is among the least characterized, being predominantly expressed in the human cornea, and elevated in the aqueous humor of the eye in patients with glaucoma, a disease of increased intraocular pressure (45–49). In the rat model, we observed that the number of Angptl7 RNA detections increased markedly over time and was highly correlated with total necrotic area, dilated vessels, CTCs, and lung metastases (*SI Appendix*, Fig. S5 *A*–*E*). Given that we observed strong spatial and temporal association between Angptl7 expression and the necrotic core, we next asked whether Angptl7 is required for necrotic core formation and metastatic dissemination. To answer this question, we transduced 4T1 tumor cells with a puromycin selectable membrane-GFP lentiviral vector and a second blasticidin selectable lentivirus encoding for shRNA hairpin and expressing cytoplasmic mCherry to generate stably transduced nontargeting control and Angptl7 knockdown (KD) lines expressing both fluorescent reporters (Fig. 3*A*). No differences in survival, proliferation, or ability to form clusters were observed between conditions during in vitro culture. Nontargeting control knockdown lines and Angptl7-knockdown (KD1-3) were each transplanted orthotopically into SRG rats. Because *Angptl7* expression was undetectable in vitro (*SI Appendix*, Fig. S4*E*), but highly induced in vivo, knockdown efficiency was confirmed in harvested tumor. By qPCR, Angptl7 RNA expression was markedly reduced with all three knockdown KDs (Fig. 3*B*). Because Angptl7 expression is low in whole tumor lysates, knockdown was confirmed on a protein level by ELISA of tumor interior, and Angptl7 protein expression was significantly reduced in KD1 and KD3 in the tumor interior, where Angptl7 expression is higher than in whole tumor lysates (Fig. 3*C* and *SI Appendix*, Fig S6*A*).

Having confirmed knockdown, we next evaluated tumor histology by H&E staining. Strikingly, knockdown of Angptl7 induced between 53 and 77% reduction in the area of necrotic cores (Fig. 3 *D* and *E*). One possibility is that decreasing necrosis could promote tumor growth, a tradeoff observed in prior studies with perturbations improving vascularization (10, 50). However, suppression of Angptl7 did not increase either total viable area, tumor weight, or tumor volume, indicating that Angptl7 suppression does not promote tumor growth (Fig. 3 *F* and *G* and *SI Appendix*, Fig. S6*B*).

**Fig. 4.**
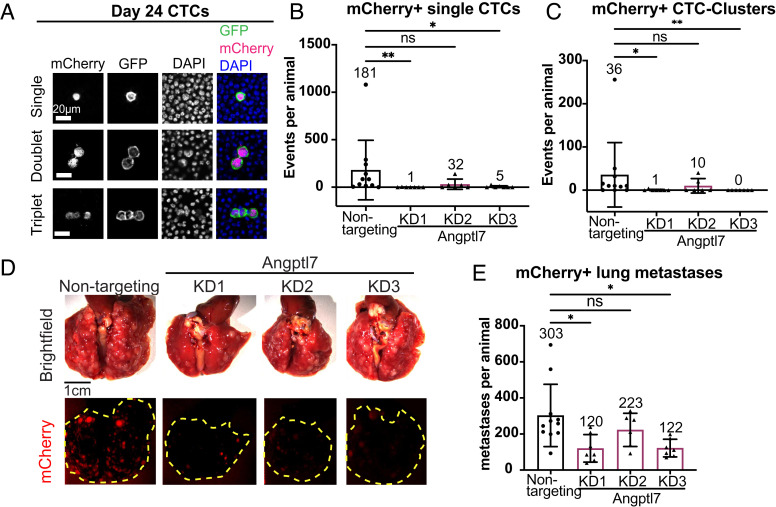
Suppression of Angptl7 reduces CTC abundance and distant lung metastases. (*A*) Representative images of single CTCs and CTC clusters from nontargeting control. Cells are mCherry-positive if they express shRNAs. 4T1 cells are labeled by membrane GFP. DAPI marks nuclei. (*B* and *C*) mCherry-positive single CTC and CTC cluster abundance in Angptl7 knockdown and nontargeting control. (*D*) Representative images of lung metastases from Angptl7 knockdown or nontargeting control transplantation (4T1 cells). Cells expressing the shRNAs express mCherry. (*E*) mCherry-positive lung metastasis count for Angptl7 knockdown and nontargeting control. Mean ± SD. All graphs shown display mean ± SD. Mean values shown on graphs. *P*-values for *B*, *C*, and *E* determined by ANOVA. *P <0.05, **P < 0.01, ***P < 0.001, and ****P < 0.0001.

Given these effects on the histology of the tumor core, we next evaluated the effect of Angptl7 knockdown on CTC abundance and metastasis. Remarkably, we observed drastic reductions in both mCherry+ single-CTC and CTC-cluster abundance, which reduced from an average of 181 single CTCs down to 1 single CTC, and from 36 CTC-clusters down to 0 or 1 CTC-clusters for the best knockdowns (Fig. 4 *A*–*C* and *SI Appendix*, Fig. S6*C*). Likewise, the number of mCherry+ lung metastases was also markedly reduced in Angptl7 knockdown conditions (Fig. 4 *D* and *E*). For the different knockdowns, the degree of necrosis suppression correlated with degree of CTC dissemination and metastasis suppression. Likewise, we observed reductions in GFP+ CTC, CTC cluster, and lung metastasis abundance (*SI Appendix*, Fig. S6 *D–F*). Taken together, these data establish that Angptl7, a tumor-specific factor that is specifically expressed in the tumor interior, is necessary for necrosis formation in the tumor core, dissemination of both single and clustered CTCs, and lung metastasis.

### Angptl7 Suppression Normalizes a Subset of Tumor Core Gene Expression and Regulates Blood Vessel Morphology and Vascular Permeability.

Given the marked decrease in intratumoral necrosis, CTC abundance, and metastasis when Angptl7 was suppressed, we next transcriptionally characterized Angptl7 knockdown tumors. Bulk RNA-seq was performed, tumor core and rim were compared, and tumor and host-derived transcripts were deconvoluted. We applied tumor core and rim gene sets from our initial xenograft deconvolution experiment (Fig. 2*A*), evaluated gene set enrichment between conditions, and observed marked depletion of tumor-derived core and host-derived core gene expression in both core and rim of Angptl7 knockdowns (*SI Appendix*, Fig. S7*A* and Table S3). Likewise, we observed marked enrichment for tumor-derived rim and host-derived rim gene expression in both core and rim of Angptl7 knockdowns.

While depletion of core gene expression was in line with the significant reduction in necrosis observed by H&E, this depletion was not complete, with 23% of tumor derived core genes and 35% of host derived core genes increased in Angptl7-knockdowns (*SI Appendix*, Fig. S7*B*). Gene ontology analysis was performed to stratify the tumor core program into normalized and nonnormalized components. Tumor-derived blood vessel morphogenesis genes and host-derived neutrophil degranulation gene sets were depleted in Angptl7-knockdown conditions, while core matrisome (tumor), RNA translation (host), and TCA transport (host) persisted or increased in Angptl7-knockdown condition compared with nontargeting control (*SI Appendix*, Fig. S7*C*). Taken together, these transcriptional studies reveal that Angptl7 regulates large-scale remodeling of the tumor core. Angptl7 supports expression of a subprogram of genes expressed in the tumor core that are involved in blood vessel morphogenesis and neutrophil degranulation. However, Angptl7-knockdown does not correct tumor core defects in RNA and nutrient metabolism, indicative of persistent cellular stress in the tumor interior.

**Fig. 5.**
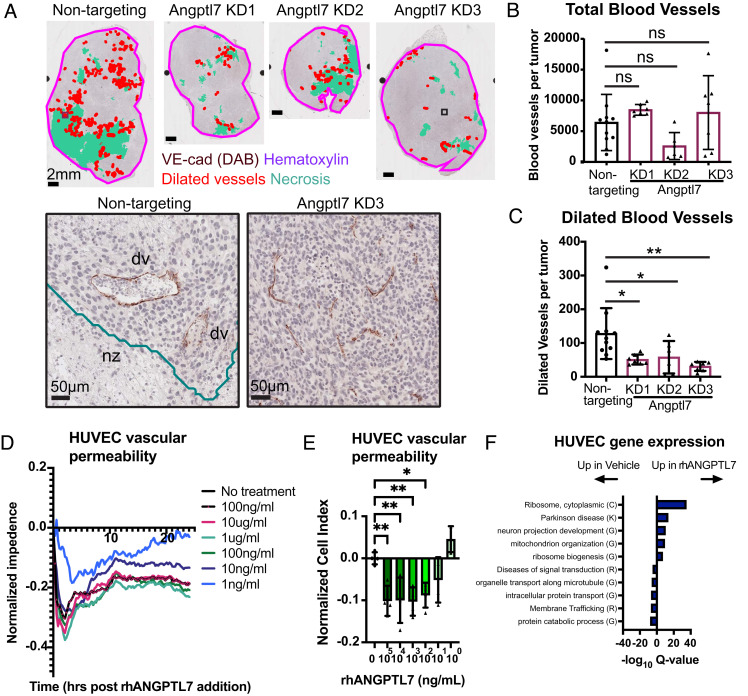
Angptl7 regulates blood vessel morphology and vascular permeability. (*A*) Representative images of dilated VE-cadherin+ blood vessels Angptl7 knockdown and nontargeting control tumors. Immunohistochemistry for VE-cadherin (DAB) (brown) counterstained with hematoxylin. Red: dilated vessels, turquoise: necrotic region, pink: tumor border. (*Insets*): vessel morphology in nontargeting control and Angptl7 knockdown. (*B*) Number of total VE-cadherin+ blood vessels in the Angptl7 knockdown and nontargeting control tumors. Mean ± SD. (*C*) Number of dilated VE-cadherin+ blood vessels in the Angptl7 knockdown and nontargeting control tumors. Mean ± SD. (*D* and *E*) Human endothelial cell (HUVEC) vascular permeability in response to ANGPTL7 recombinant protein (rhANGPTL7). Vascular permeability was measured as normalized impedance over time. n = 4 biological replicates. Shown is mean temporal response (*D*) and normalized cell index at 1 h (*E*). (*F*) Metascape analysis of gene enrichment. HUVEC cells were treated with rhANGPTL7 for 24 h. RNA-seq identified 741 up-regulated genes and 500 genes down-regulated with rhANGPTL7 treatment with *P*-value cutoff ≤ 0.01. Enrichments reported as -log Q-values. All *P*-values determined by one-way ANOVA. *P <0.05, **P < 0.01, ***P < 0.001, and ****P < 0.0001.

Having identified vascular morphogenesis genes as potentially Angptl7 regulated, we next characterized the spatial distribution and expression of blood vessels in the Angptl7 knockdown tumors (Fig. 5*A*). Total VE-cadherin-positive vessel number and density were similar between Angptl7 knockdown and nontargeting control tumors (Fig. 5*B* and *SI Appendix*, Fig. S8 *A* and *B*). In contrast, the number and density of VE-cadherin-positive dilated vessels were markedly reduced in Angptl7 knockdown tumors (Fig. 5*C* and *SI Appendix*, Fig. S8*B*). Further, smooth muscle actin staining, indicative of smooth muscle cell or contractile pericyte coverage, was uncommon around dilated vessels in both control and Angptl7 knockdown tumors, and not altered by Angptl7 suppression (*SI Appendix*, Fig. S8 *C*–*F*). Because Angptl7 is also reported to regulate lymphangiogenesis (40), we also stained lymphatics for podoplanin. In contrast to VE-cadherin, we observed no significant difference in lymphatic vessel number, morphology, or perinecrotic localization (*SI Appendix*, Fig. S9 *A*–*D*). Lymphatic vessels were more prominent in viable regions of the tumor, closer to the tumor edge (*SI Appendix*, Fig. S9 *C* and *D*). Taken together, these data show that Angptl7 is necessary for the perinecrotic dilated vessel phenotype.

Given the effect of Angptl7 suppression on dilated vessel morphology, we next examined the effect of ANGPTL7 on vascular permeability. Human umbilical endothelial vein cells (HUVECs) plated on 2D were incubated with recombinant human ANGPTL7 from 1 ng/mL to 100 μg/mL and measured for changes in vascular permeability using a live-cell impedance assay. Strikingly, HUVECs showed a dose-dependent response to ANGPTL7 addition that was most pronounced by 1 h of treatment, which persisted out to 24 h at 10 ng/mL and higher recombinant ANGPTL7 concentrations (Fig. 5 *D*–*F*).To define gene pathways associated with ANGPTL7-induced changes, HUVEC cells were treated with 10 μg/mL ANGPTL7 for 24 h and then harvested for RNA-seq. Gene ontology analysis revealed dominant enrichment for cytoplasmic ribosome-encoding genes and weaker enrichment for genes involved in Parkinson disease, neuron projection development, and mitochondrial organization (Fig. 5*F* and *SI Appendix*, Table S4). Signaling pathway enrichment was observed for genes involved in SLIT and ROBO signaling (log Q-value −32), previously implicated in endothelial-tumoral crosstalk during metastatic dissemination (51). Taken together, these data indicate that Angptl7 supports vascular remodeling in vivo, increases vascular permeability, and induces gene expression changes in endothelial cells in vitro.

### *ANGPTL7* is Highly Expressed in High-Necrosis Triple-Negative Human Breast Cancer Patient-Derived Xenografts (PDXs).

**Fig. 6.**
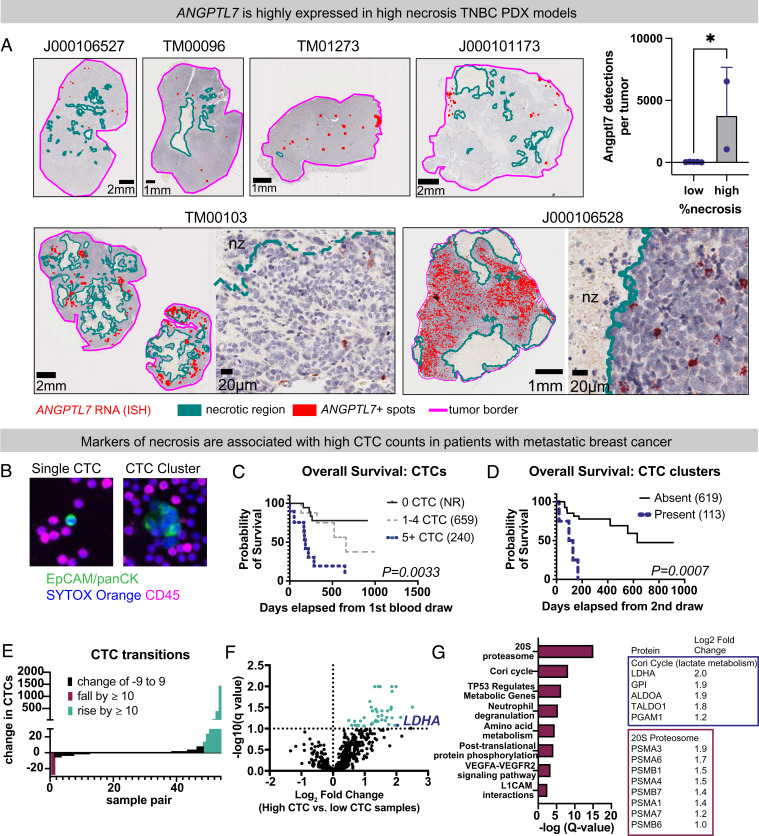
*ANGPTL7* is expressed in high necrosis human triple-negative breast cancers and necrosis markers correlate with CTC dissemination in breast cancer patients. (*A*) *ANGPTL7* RNA ISH performed on tumor sections from human breast PDX tumors transplanted into NSG mice. All tumor models were invasive ductal carcinomas and confirmed triple-negative for ER, PR, and HER2 on PDX tumor sections. Qupath detections shown. Turquoise: necrotic region, red: *ANGPTL7*-positive detections, pink: tumor border. (*Insets*) show raw *ANGPTL7* RNA ISH in red. (*Right*) Bar graph comparing tumors with low necrosis (five sections from four models) and high necrosis (two sections from two models). High necrosis defined as >15% necrosis by area. *P*-value determined by unpaired *t* test. (*B*) Representative single CTC and CTC cluster from clinical vignette in *SI Appendix, *Fig. S6. EpCAM/pan-CK, epithelial cells; CD45, immune cells; SYTOX Orange, nucleic acid stain. (*C*) Overall survival according to CTC enumeration at baseline. Median overall survival reported in parentheses. NR = not reached. *P*-value determined by Mantel–Cox log-rank test. (*D*) Overall survival according to presence or absence of CTC-clusters. Landmark analysis from 2nd blood draw was performed. Median overall survival reported in parentheses. *P*-value determined by Mantel–Cox log-rank test. (*E*) Waterfall plot showing changes in CTC abundance between time points, sorted by magnitude. (*F*) Tandem-mass tag mass spectrometry of high vs. low-CTC samples. Volcano plot shows the 46 plasma proteins enriched in three high-CTC samples compared with 13 low CTC samples. Proteins with Q-values less than 10%. False discovery rate determined by two-stage set-up method by Benjamini, Krieger, and Yekutieli. (*G*) Metascape analysis of CTC-associated proteins reported by -log Q-value. (*Right*) Proteins with fold-enrichment that make up core enrichments for Cori cycle and 20S proteasome. *P <0.05, **P < 0.01, ***P < 0.001, and ****P < 0.0001.

Clinically, necrotic zones are generally avoided during diagnostic tissue sampling due to lower abundance of viable tissue in these regions. To extend the human disease relevance of our findings in rat models, we evaluated *ANGPTL7* mRNA by RNA-ISH from seven tumor sections from six different human breast cancer patient-derived xenografts (PDXs) each derived from high-grade invasive ductal carcinomas, and each triple-negative (ER-negative, PR-negative, HER2-negative receptor status) by PDX tumor histology (Fig. 6*A*). Even among these high-grade tumors, the percentage of necrosis varied markedly between models. Likewise, *ANGPTL7* expression varied between models and was markedly higher in high necrosis models. For comparison, model J000106527, which had very low necrosis and *ANGPTL7* expression was derived from a patient with T4bN0 high-grade triple-negative breast cancer (TNBC). This patient developed a second primary breast cancer 4 years later that was resected and then treated with adjuvant endocrine therapy. In contrast, model J000106528, which had the highest necrosis and *ANGPTL7* expression of all models tested, was derived from a patient with T3N2 high-grade TNBC. This patient developed metastatic breast cancer within 2 years from initial diagnosis and tumor resection and progressed rapidly on therapy. Taken together, these clinical observations and experimental studies in PDX models demonstrate that ANGPTL7 is associated with TNBCs with large necrotic zones.

### Necrosis Markers Are Associated with CTC Dissemination and Metastasis in Breast Cancer Patients.

Given the association between necrosis and CTC abundance in our rat model, we hypothesized that necrosis markers are associated with CTC dissemination in human breast cancer patients. To test this hypothesis, we interrogated longitudinal CTC abundance from patients with metastatic breast cancer recruited to an IRB-approved study at the Fred Hutchinson Cancer Center and the University of Washington (FH 8649). Over a 3 y period, a total of 102 blood samples from 40 patients were longitudinally collected including 35 women with estrogen receptor-positive metastatic tumors and five with estrogen receptor status that was negative or unknown (See *SI Appendix*, Table S5 for demographic information). For each blood sample, the cellular fraction was separated by density, deposited to slides, and enumerated for CTCs by computer-assisted image analysis using the Rarecyte platform. Twenty nine patients (73%) had at least one CTC at any timepoint, five patients (13%) had at least one CTC cluster at any timepoint, and 11 patients (28%) had no CTCs at any timepoints. As expected based on prior studies, patients with CTCs detected at first blood draw had significantly worse overall survival and early death (Fig. 6 *B*–*C*). Likewise, patients with CTC clusters at first or second blood draw had markedly worse overall survival and early death (Fig. 6 *B* and *D*).

Among patients with CTCs at any time point, substantial differences in CTC dynamics were observed, and in a subset of cases, extreme elevations in CTC abundance between time points (Fig. 6*E* and *SI Appendix*, Fig. S10 *A* and *B* for case vignette). Sixteen blood samples were selected from eight patients, made up of matched early and late time-points (*SI Appendix*, Table S6). Blood plasma from all 16 samples were depleted of high-abundance proteins, labeled with tandem-mass-tags, and interrogated by LC-ESI mass spectrometry. Comparison of high-CTC samples with all low-CTC samples yielded 46 proteins highly associated with high-CTC state and passing q-values of 0.1 or less (Fig. 6*F*). ANGPTL7 was not detected by LC-ESI mass spectrometry precluding its comparison between CTC states. Metascape gene set analysis demonstrated the top enrichment being for gene sets linked to necrosis (Fig. 6*G*) including: 1) Cori cycle, the major pathway for lactate metabolism, 2) the 20S proteosome which has been associated with necrosis, and 3) neutrophil degranulation, which we also identified as a top gene set in our core/rim RNA-seq analyses (52, 53). To control for interpatient variation, we further conducted a pair-wise comparison of late vs. early time points from three patients with low-to-high CTC transitions. Of the 17 proteins increased twofold or more between late and early time points, 13 (76%) were necrosis associated (*SI Appendix*, Fig. S10*C*). To validate our proteomic observations, we further measured levels of the necrosis associated marker lactate dehydrogenase (LDH) from plasma for 93 blood samples from 39 patients. Consistent with our proteomic enrichments, marked elevations in LDH were associated with a 10 or more increase in CTC abundance between time points, with blood samples with 20 or more CTCs, and with blood samples with CTC-clusters (*SI Appendix*, Fig. S10 *D*–*F*). Taken together, these clinical, biochemical, and proteomic studies show that as seen in our rat model experiments, low-to-high CTC transitions are associated with increasing CTCs and markers of necrosis in patients with metastatic breast cancer.

## Discussion

A key challenge in metastasis research is to discern where and how tumor cells disseminate to distant organs. However, understanding where metastatic dissemination originates from is a formidable challenge. Tumor dissemination is a dynamic process that is difficult to capture (17, 30–35), and occurring in the context of spatially heterogeneous ecosystems varying in their nutrient availability, perfusion, oxygenation, and host contributions (12, 54, 55). Here, we developed a rat transplantation model of breast cancer that increases CTC detection 10-fold and leveraged this model to identify cellular and molecular changes in primary tumor associated with tumor dissemination. Importantly, we observed that tumor dissemination was strongly correlated temporally with necrosis in animal models and in human cancer patients, that tumor dissemination was localized spatially to dilated perinecrotic vessels in the tumor interior, and that tumor dissemination was dependent functionally on the expression of a factor, Angptl7, produced by perinecrotic tumor cells. Our findings in breast cancer models, in conjunction with recent clinical observations (25, 29), provide strong evidence for tumor dissemination from the tumor interior.

Decoding the molecular regulation of dissemination in the tumor interior is a particularly challenging chicken-and-egg problem because the tumor core is composed of regions that are hypoxic, deficient in nutrients, and abnormally perfused (12, 54, 55), each of which could influence and in turn be influenced by adaptive responses from tumor cells in the core (12). The very large tumors produced in our rat model enabled us to spatially dissect tumor core from tumor rim for transcriptional profiling, and the use of xenograft deconvolution methods further enabled us to discern which genes were expressed specifically in tumor and host. In this regard, the use of a mouse-in-rat xenograft was serendipitous and essential. We suggest that rats are a valuable model organism with unique attributes supporting their use in metastasis research alongside more commonly used in vivo models such as mouse and zebrafish. These studies unmasked a tumor-derived program in the core and identified Angptl7 as the top-ranked secreted factor. Strikingly, Angptl7 suppression normalizes histologic findings of central necrosis and dilated perinecrotic vasculature. On a molecular level, Angptl7 depletion resolves vascular morphogenetic, hypoxic, and inflammatory changes associated with the tumor core but not gene programs associated with stress, RNA metabolism and nutrient depletion. These findings suggest that tumor necrosis arises from the sequential activation of stress programs in the core that induce a tumor-cell-driven transcriptional response including Angptl7. Thus, Angptl7 is both regulated by the local microenvironment in the tumor interior and a regulator of further central necrosis and metastatic dissemination. Future studies are needed to define the signals and regulatory programs driving the tumor core adaptive response program.

Previous studies have shown that tumor dissemination can depend on macrophage-led migration, vascular mimicry, EMT, and collective migration (6, 7, 56–64). Our data establish the importance of the necrotic zone in supporting metastatic dissemination and highlight the presence of intravascular tumor emboli within dilated perinecrotic vessels. We note that both dilated vessels and Angptl7 were on average 2 mm away from the nearest tumor border. Given the great distance of the central necrotic zone to the tumor-stromal interface, advances in intravital imaging in deep tissues (65, 66) will be essential to evaluate which of these cellular dissemination mechanisms predominates in the necrotic zone. In addition, organotypic culture models have proven useful in modeling invasion in vitro that have morphologic correlates in vivo (61, 62, 67, 68). Our transcriptional dissection provides a reference point in which to develop organotypic models that more faithfully mimic the necrotic core-associated dissemination program. In particular, the formation of dilated vessels is strongly associated with tumor dissemination and metastasis. At present, how vessels become dilated is unclear. Whether dilated vessels arise from extrinsic compression from proliferating tumor cells, influence of the extracellular matrix, or reflects occlusion by tumor cell emboli is unclear and could benefit from better organotypic models incorporating vasculature. Finally, previous clinical and experimental studies have shown that tumors can disseminate early preceding the detection of frank invasive cancer (69, 70), while at the same time, that very large tumors are more likely to develop metastatic cancer (71, 72). Our data are compatible with these clinical observations. Because tumor necrosis can arise independently of tumor invasion, small fast-growing tumors could undergo necrosis early, while large tumors could undergo necrosis late. Consistent with our experimental observations, comedonecrosis is associated with increased risk of death from in situ, preinvasive cancers (73).

From a therapeutic standpoint, our data indicate that ANGPTL7 is a fulcrum for eliciting central necrosis and metastatic dissemination. ANGPTL proteins have three conserved domains including an N-terminal coiled-coil domain that mediates homo-oligomerization, a linker peptide, and a C-terminal fibrinogen-like domain (74). More studies are needed to investigate the functional domains of ANGPTL7 necessary for necrosis and metastatic dissemination. Therapeutic blocking antibodies and anti-sense oligonucleotides targeting ANGPTL3 reduce atherogenic lipoprotein levels and decrease the odds of developing cardiovascular disease in humans (38, 39), supporting ANGPTL family of proteins as druggable targets. Necrosis is associated with aggressive tumors and is a common tumor response to many cancer treatments. Given the pronounced effects of Angptl7 depletion in our rat model, we suggest that ANGPTL7 is a target for future drug development for necrosis and metastasis suppression.

## Materials and Methods

### Experimental Model and Subject Details.

#### Animal models.

All mice were maintained under specific pathogen-free conditions, and experiments conformed to the guidelines as approved by the Institutional Animal Care and Use Committee of Fred Hutchinson Cancer Research Center (FHCC).

#### Human breast cancer patient samples.

Blood from patients were obtained from consenting patients under a Fred Hutch IRB approved study (FH8649) for longitudinal monitoring of circulating tumor cells in metastatic breast cancer patients. CTCs and CTC-clusters were enumerated in these fluid samples using a RareCyte assay (75). For details on the receptor status and pathology of each deidentified human sample used, see *SI Appendix*, Table S1.

### Methods.

#### Generating knockdown 4T1 cell lines.

4T1-AcGFP1-mem-9 cells were transduced with lentiviral particles generated with shERWOOD UltramiR Lentiviral Inducible shRNA (TransOMIC) for Angptl7 and selected with 10 ug/mL puromycin + 10 ug/mL blasticidin for at least 2 weeks.

#### Orthotopic transplantation into mammary fat pad of rats and mice.

Cells to be transplanted were cultured 3D for two passages before being transplanted. After the second passage, cells were plated in nonadherent six-well plates at 150 k cells/mL with 4 mL media per well for the cells to aggregate over 24 h before being transplanted. Spheroids formed this way were resuspended in 1:1 Matrigel:DMEM/F12 mix, kept on ice. A total of 600,000 cells in 20 µL Matrigel DMEM/F12 mix were transplanted into the right T4 mammary fat pad of rats or mice. Estimated tumor volume was calculated based on caliper measurements with the formula: V = (W^2^ × L)/2.

#### Density separation of coat (nucleated cells) from blood.

Peripheral blood was diluted 1:1 with D-PBS then layered on top of 8 mL Ficoll (GE Ficoll Paque Plus GE17-1440-02 for human blood and Ficoll Paque Premium GE17-5442-02 for mouse and rat blood) in a 2.5% BSA-coated 50 mL conical tube. Tubes with Ficoll and diluted blood were centrifuged at 400 g for 35 min with 0 acceleration and deceleration. The cloudy, white layer (“buffy coat”) between the clear Ficoll layer and the top plasma layer and the plasma layer were collected into a separate BSA-coated tube. Buffy coat and plasma layer mix were centrifuged at 4 °C at 3,500 g for 30 min to pellet nucleated cells, then resuspended with D-PBS. To visualize Buffy coat containing CTCs, cell suspension was spun onto slides using a Cytospin (800 g for 5 min). After drying slides completely, slides were fixed with 4% PFA for 5 min, washed with D-PBS 5 min three times. Slides were dried completely again before being stored in −80 °C. For plasma collection, plasma layer was collected from the plasma layer from the Ficoll density separation or from a separate blood tube in an AccuCyte Blood collection tube processed by Rarecyte with a 3,000 g 25 min spin at 25 °C.

#### RNA-seq computational deconvolution of mouse and rat genomes.

RNA-seq reads in xenograft samples were deconvolved for identification of the species of origin following a method similar to that described by Wingrove et al. (72). See *SI Appendix*, *Supplementary Methods* for more detail.

#### QuPath quantification of RNA ISH.

RNAscope images were opened in QuPath (76). Thresholds were created to identify tumor area and necrotic area by eye, verified to be accurate in test images, and applied to demarcate necrotic zones. Angptl7-expressing cells were identified using positive cell detection. The distance to annotation 2d feature was used to determine the distance between cells and the nearest necrotic border and cells and nearest tumor border. This process was automated in QuPath for consistent analysis across images.

#### Necrosis measurements.

Tumor necrosis measurements were determined based on hematoxylin & eosin (H&E) or 2,3,5-triphenyltetrazolium chloride (TTC) assay. For H&E staining, tumors were sliced into two to three ~5 mm slices by scalpel, fixed for 5 d in 10% formalin at 4 °C, rocking before being sectioned, and stained with hematoxylin and eosin. For the TTC assay, tumors were sliced into two to three ~5 mm slices and stained with 1 g/100 mL tetrazolium salt in a 7.4 pH buffer with 77.4% NaH2PO4 (0.1 M) and 22.6% Na2HPO4 (0.1 M) mix, at 37 °C for 20 min, rocking. Tumor slices were then fixed with 10% formalin for 20 min before being visualized. In the TTC assay, the TTC compound is reduced to a red TPF (1,3,5-triphenylformazan) compound in live tissues due to dehydrogenase activity. White areas therefore indicate necrotic tissue, and red areas are viable regions.

### Quantification and Statistical Analysis.

Bars are presented as mean ± SD. Graphs were created and statistical tests conducted in GraphPad Prism 8. Nonparametric tests were used when data were not normally distributed or when the median was a better representation of the sample than the mean. For animal experiments, each animal was considered a biological replicate. For in vitro experiments, experiments using cell lines on different days were considered biological replicates. *P*-values were denoted as follows: *P < 0.05, **P < 0.01, ***P < 0.001, and ****P < 0.0001.

## Supplementary Material

Appendix 01 (PDF)

Dataset S01 (XLSX)

Dataset S02 (XLSX)

Dataset S03 (XLSX)

Dataset S04 (XLSX)

Dataset S05 (XLSX)

Dataset S06 (XLSX)

## Data Availability

Raw RNA-sequencing data have been deposited on the National Center for Biotechnology Information (NCBI) Sequence Read Archive (SRA) (BioProjects: PRJNA925873 (77), PRJNA925929 (78), PRJNA928719 (79); for necrotic core vs. rim, Angptl7 KD vs. NT, and HUVEC Angptl7 treatment vs. control, respectively). RNA-seq analysis code: The RNA-seq analysis code used to analyze tumor xenograft samples are available on Zenodo: DOI: 10.5281/zenodo.7574251, and future versions will be maintained on github: https://github.com/bkrajina/RNA_seq_xenograft_analysis. Qupath spatialanalysis code: DOI: 10.5281/zenodo.7574395.
