# Supplementary material for: Metastasis from the tumor interior and necrotic core formation are regulated by breast cancer-derived angiopoietin-like 7
Source: Proc Natl Acad Sci U S A. 2023 Feb 28;120(10):e2214888120. doi: 10.1073/pnas.2214888120 (PMC10013750; doi:10.1073/pnas.2214888120)
Supplement: Supplementary file 1 — Appendix 01 (PDF) [file pnas.2214888120.sapp.pdf]

PNAS

Supporting Information for
Metastasis from the tumor interior and necrotic core formation are regulated by breast cancer-derived angiopoietin-like 7

Ami Yamamoto, Yin Huang, Brad A. Krajina, Margaux McBirney, Andrea E. Doak, Sixuan Qu, Carolyn L. Wang, Michael C. Haffner, Kevin J. Cheung.

Corresponding author:
Kevin J. Cheung
Email: kcheung@fredhutch.org

This PDF file includes:

Figure S1-S10
Legends for Figure S1-S10
Supplementary Methods
Key Resources Table
SI Appendix References

Other supporting materials for this manuscript include the following:

Supplementary Table 1- DE core vs rim
Supplementary Table 2- Metascape core vs rim
Supplementary Table 3- DE for Angptl7 KD vs non-targeting tumor
Supplementary Table 4- HUVEC rhANGPTL7 RNA-seq
Supplementary Table 5- Patient Demographics
Supplementary Table 6- Patient LCMS Samples

Figure S1

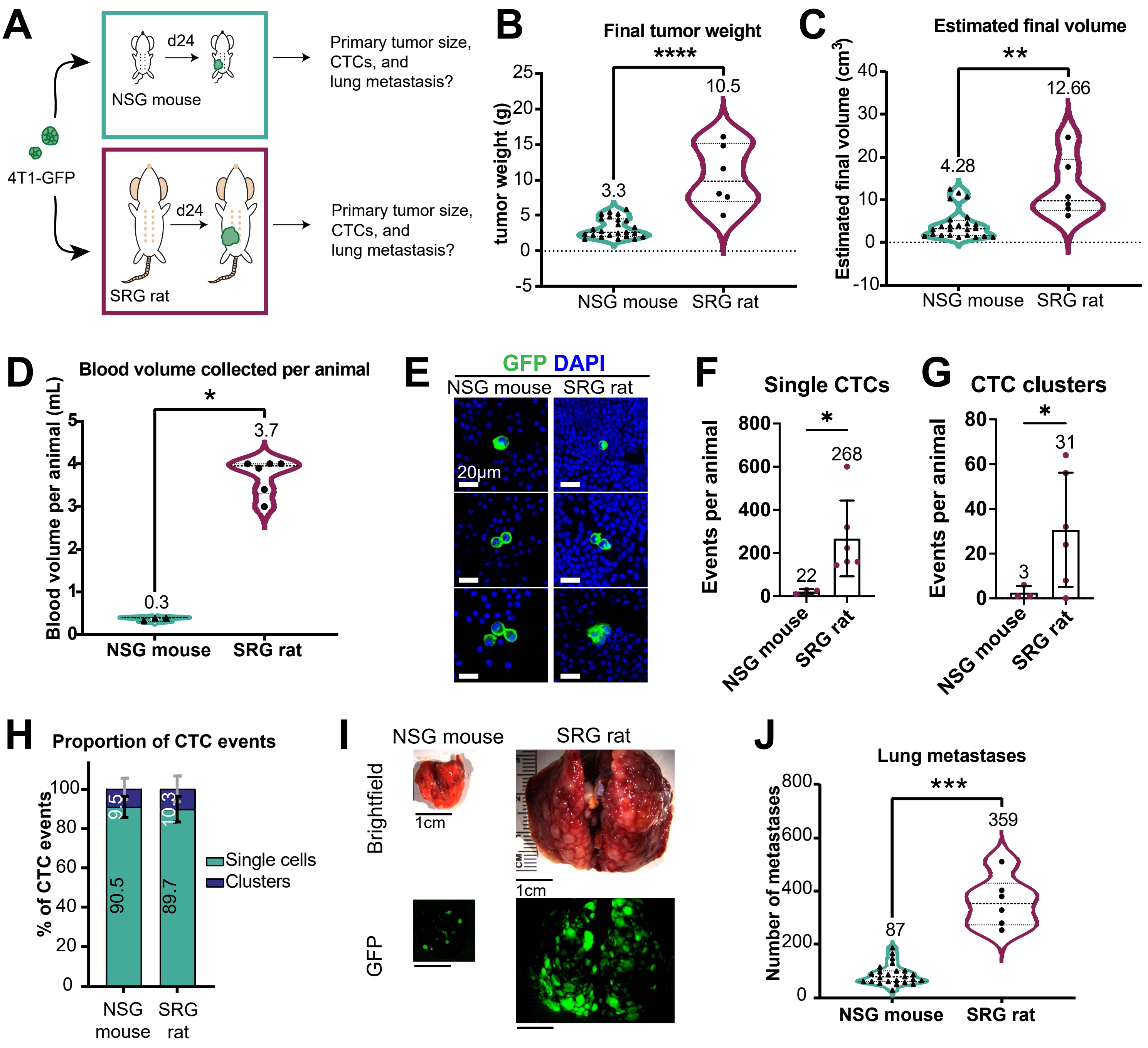

A
d24
NSG mouse
Primary tumor size,
CTCs, and
lung metastasis?
4T1-GFP
d24
SRG rat
Primary tumor size,
CTCs, and
lung metastasis?
B
Final tumor weight
****
tumor weight (g)
3.3
10.5
NSG mouse
SRG rat
C
Estimated final volume
**
Estimated final volume (cm³)
4.28
12.66
NSG mouse
SRG rat
D
Blood volume collected per animal
*
Blood volume per animal (mL)
0.3
3.7
NSG mouse
SRG rat
E
GFP DAPI
NSG mouse
SRG rat
20µm
F
Single CTCs
Events per animal
*
22
268
NSG mouse
SRG rat
G
CTC clusters
Events per animal
*
3
31
NSG mouse
SRG rat
H
Proportion of CTC events
% of CTC events
9.5
10.3
90.5
89.7
Single cells
Clusters
NSG mouse
SRG rat
I
NSG mouse
SRG rat
Brightfield
1cm
1cm
GFP
J
Lung metastases
***
Number of metastases
87
359
NSG mouse
SRG rat

Figure S2

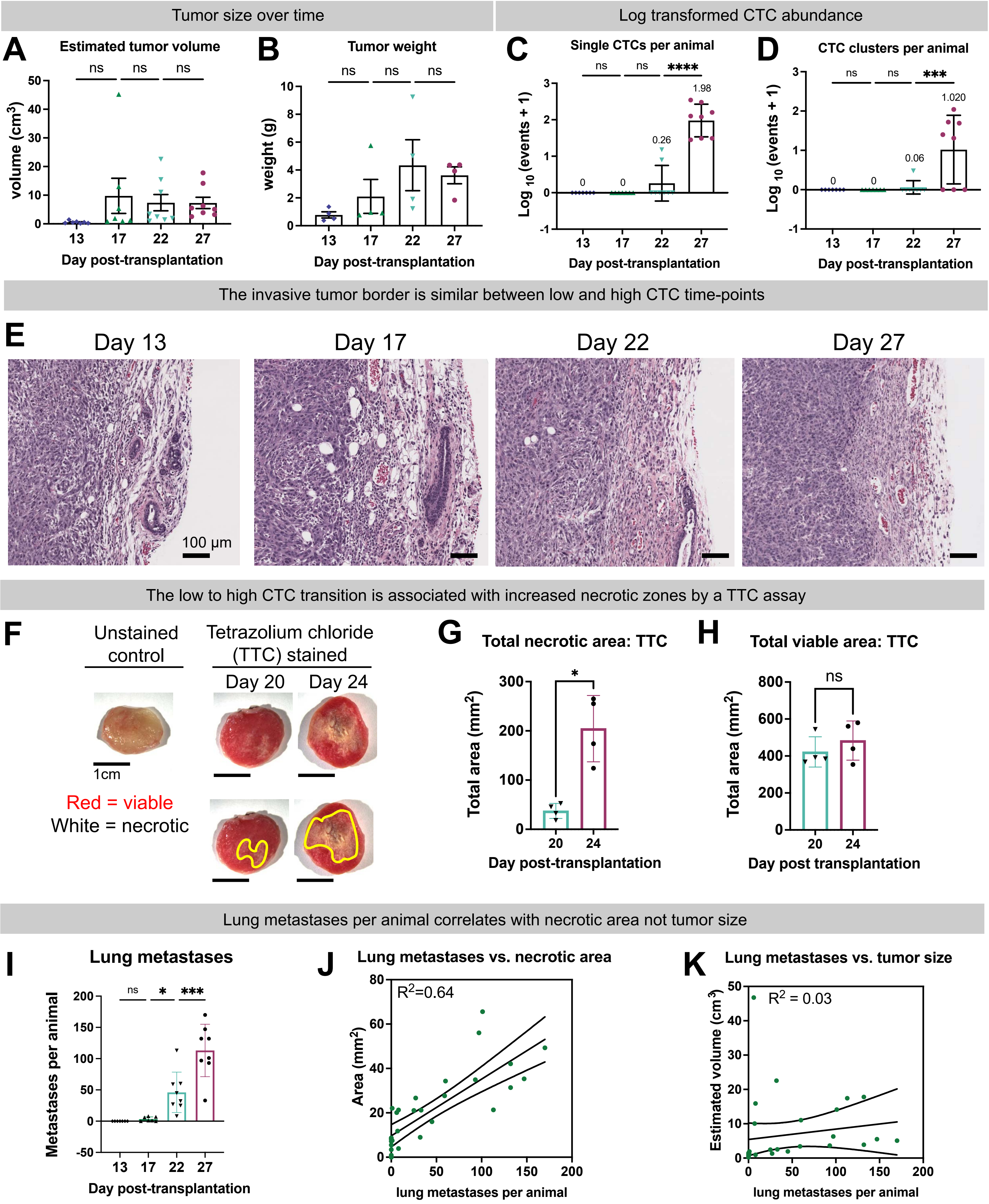

Tumor size over time
Log transformed CTC abundance
A
Estimated tumor volume
ns
ns
ns
volume (cm^3)
50
40
30
20
10
0
13
17
22
27
Day post-transplantation
B
Tumor weight
ns
ns
ns
weight (g)
10
8
6
4
2
0
13
17
22
27
Day post-transplantation
C
Single CTCs per animal
ns
ns
****
Log $_{10}$ (events + 1)
3
2
1
0
-1
0
0
0.26
1.98
13
17
22
27
Day post-transplantation
D
CTC clusters per animal
ns
ns
***
Log $_{10}$ (events + 1)
3
2
1
0
-1
0
0
0.06
1.020
13
17
22
27
Day post-transplantation
The invasive tumor border is similar between low and high CTC time-points
E
Day 13
Day 17
Day 22
Day 27
100 µm
The low to high CTC transition is associated with increased necrotic zones by a TTC assay
F
Unstained control
Tetrazolium chloride (TTC) stained
Day 20
Day 24
1cm
Red = viable
White = necrotic
G
Total necrotic area: TTC
*
Total area (mm^2)
300
200
100
0
20
24
Day post-transplantation
H
Total viable area: TTC
ns
Total area (mm^2)
800
600
400
200
0
20
24
Day post transplantation
Lung metastases per animal correlates with necrotic area not tumor size
I
Lung metastases
ns
*
***
Metastases per animal
200
150
100
50
0
-50
13
17
22
27
Day post-transplantation
J
Lung metastases vs. necrotic area
R^2=0.64
Area (mm^2)
80
60
40
20
0
0
50
100
150
200
lung metastases per animal
K
Lung metastases vs. tumor size
R^2 = 0.03
Estimated volume (cm^3)
50
40
30
20
10
0
0
50
100
150
200
lung metastases per animal

Figure S3

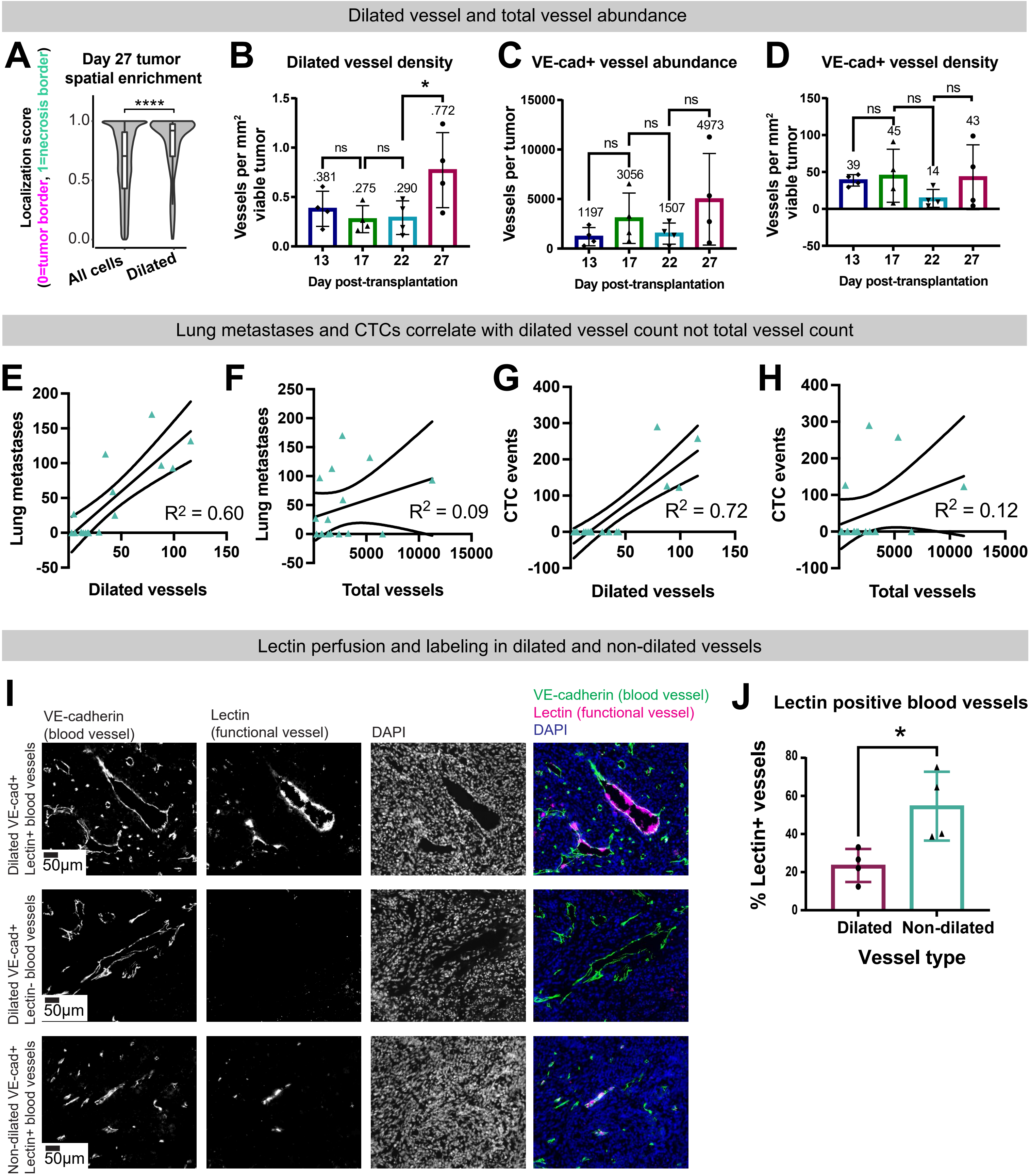
Dilated vessel and total vessel abundance
A
Day 27 tumor spatial enrichment
Localization score
(0=tumor border, 1=necrosis border)
****
1.0
0.5
0.0
All cells
Dilated
B
Dilated vessel density
Vessels per mm² viable tumor
1.5
1.0
0.5
0.0
*
ns
ns
.381
.275
.290
.772
13
17
22
27
Day post-transplantation
C
VE-cad+ vessel abundance
Vessels per tumor
15000
10000
5000
0
ns
ns
ns
1197
3056
1507
4973
13
17
22
27
Day post-transplantation
D
VE-cad+ vessel density
Vessels per mm² viable tumor
150
100
50
0
-50
ns
ns
ns
39
45
14
43
13
17
22
27
Day post-transplantation
Lung metastases and CTCs correlate with dilated vessel count not total vessel count
E
Lung metastases
200
150
100
50
0
-50
50
100
150
R² = 0.60
Dilated vessels
F
Lung metastases
250
200
150
100
50
0
-50
5000
10000
15000
R² = 0.09
Total vessels
G
CTC events
400
300
200
100
0
-100
50
100
150
R² = 0.72
Dilated vessels
H
CTC events
400
300
200
100
0
-100
5000
10000
15000
R² = 0.12
Total vessels
Lectin perfusion and labeling in dilated and non-dilated vessels
I
VE-cadherin (blood vessel)
Lectin (functional vessel)
DAPI
VE-cadherin (blood vessel)
Lectin (functional vessel)
DAPI
Dilated VE-cad+ Lectin+ blood vessels
Dilated VE-cad+ Lectin- blood vessels
Non-dilated VE-cad+ Lectin+ blood vessels
50μm
50μm
50μm
J
Lectin positive blood vessels
% Lectin+ vessels
80
60
40
20
0
*
Dilated
Non-dilated
Vessel type

Figure S4

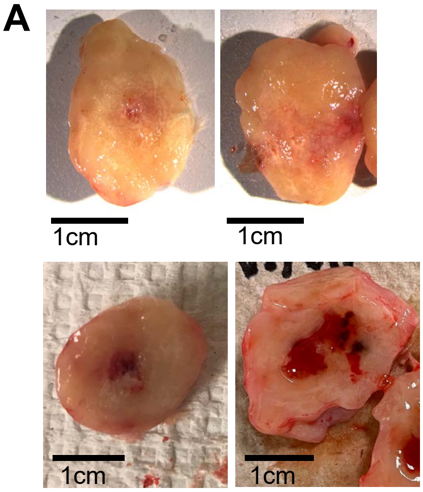

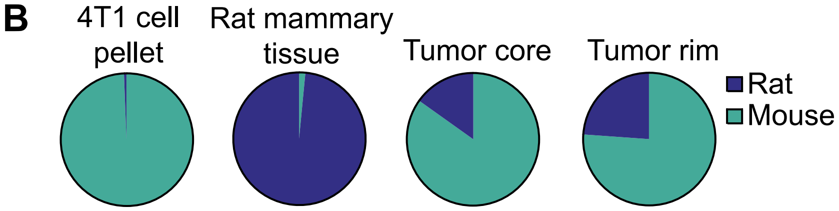

Sample Type	% mapped to mouse genome	% mapped to rat genome
4T1 cell pellet (n=1)	99.41%	0.59%
Rat mammary tissue (n=2)	1.53% ± 0.0002%	98.47% ± 0.0002%
Tumor core (n=5)	84.84% ± 1.35%	15.16% ± 1.35%
Tumor rim (n=5)	76.12% ± 5.96%	23.88% ± 5.96%

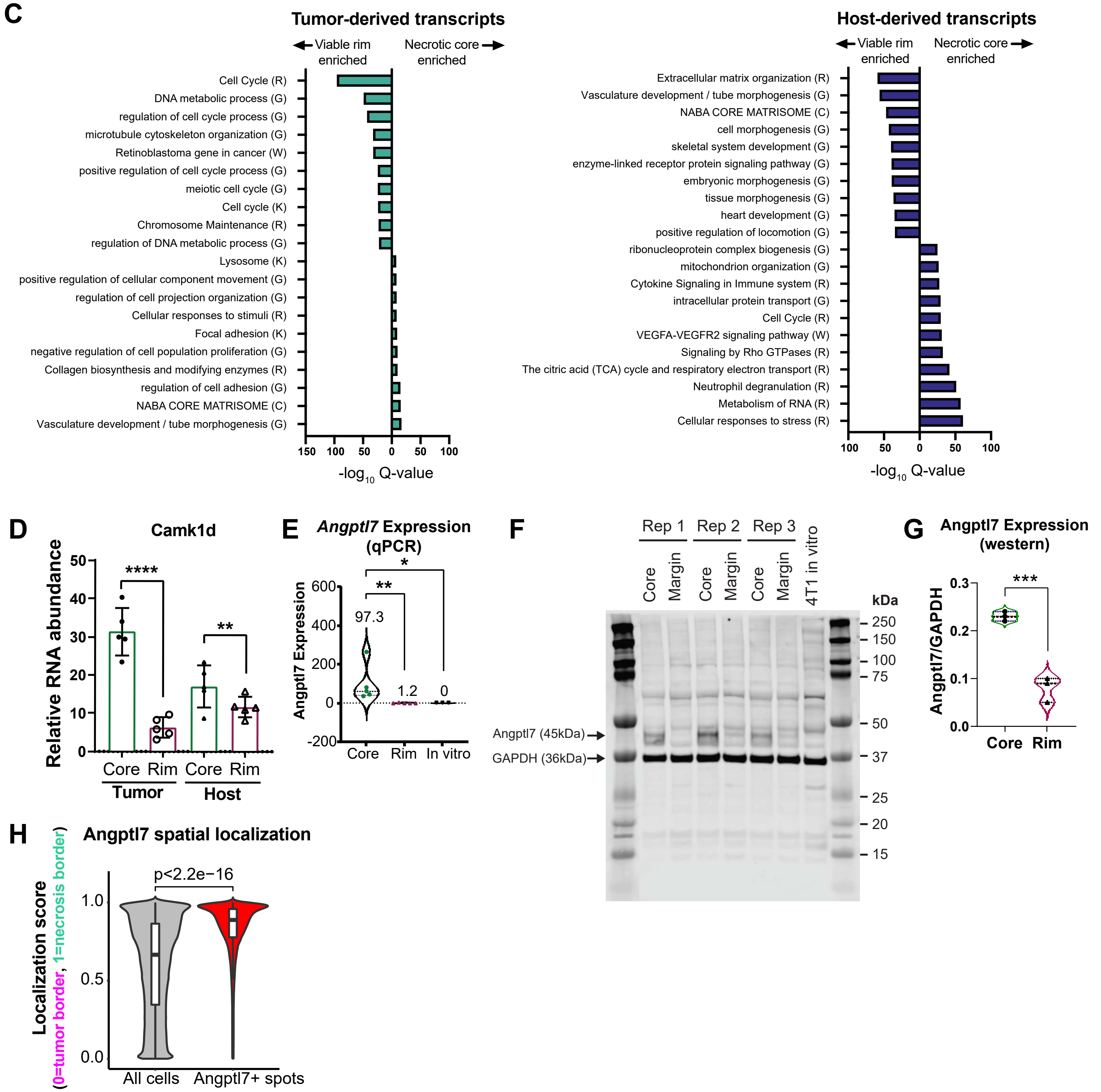

Figure S5

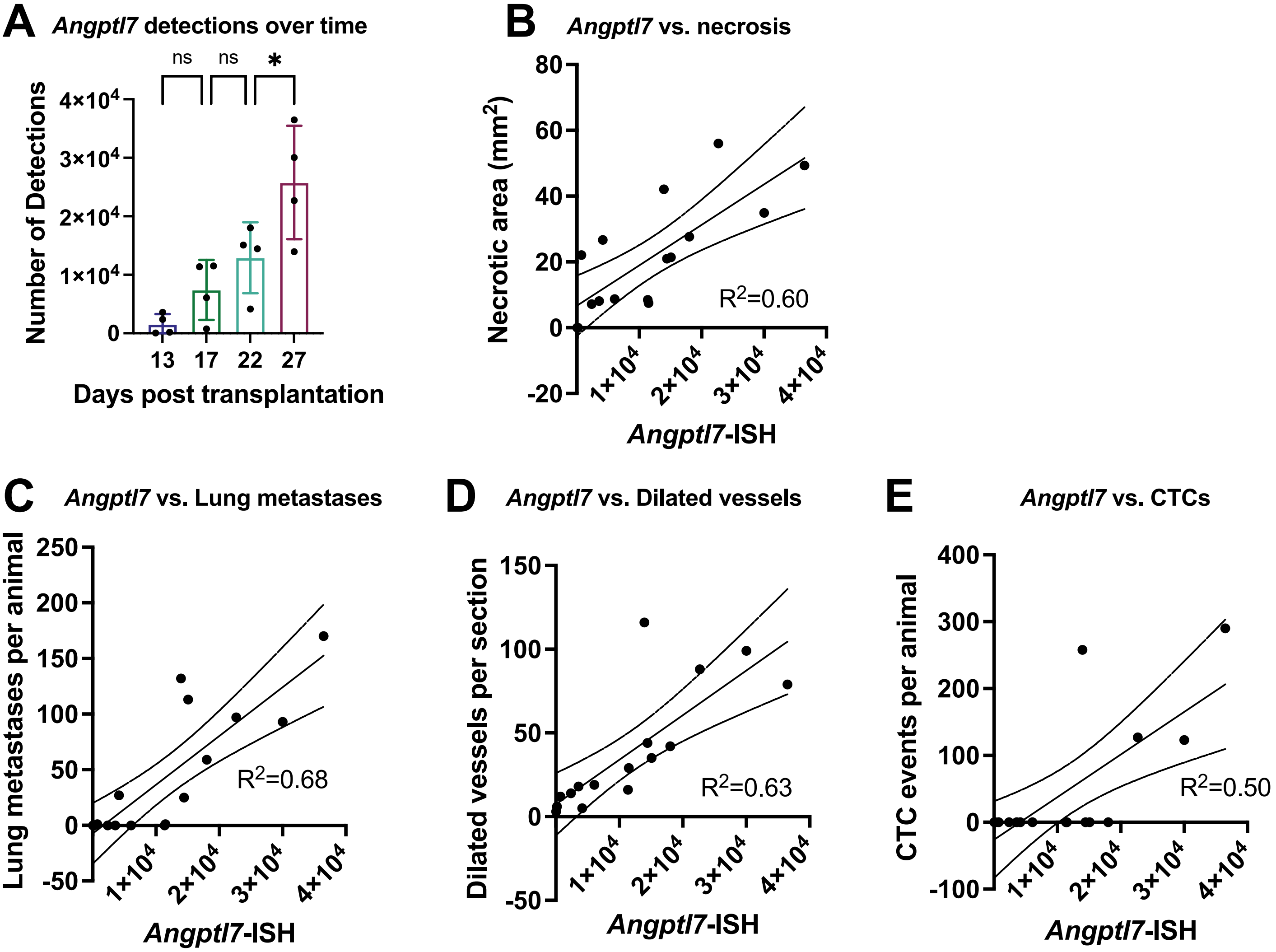

A
Angptl7 detections over time
ns
ns
*
Number of Detections
4×10^4
3×10^4
2×10^4
1×10^4
0
13
17
22
27
Days post transplantation
B
Angptl7 vs. necrosis
Necrotic area (mm^2)
80
60
40
20
0
-20
R^2=0.60
1×10^4
2×10^4
3×10^4
4×10^4
Angptl7-ISH
C
Angptl7 vs. Lung metastases
Lung metastases per animal
250
200
150
100
50
0
-50
R^2=0.68
1×10^4
2×10^4
3×10^4
4×10^4
Angptl7-ISH
D
Angptl7 vs. Dilated vessels
Dilated vessels per section
150
100
50
0
-50
R^2=0.63
1×10^4
2×10^4
3×10^4
4×10^4
Angptl7-ISH
E
Angptl7 vs. CTCs
CTC events per animal
400
300
200
100
0
-100
R^2=0.50
1×10^4
2×10^4
3×10^4
4×10^4
Angptl7-ISH

Figure S6

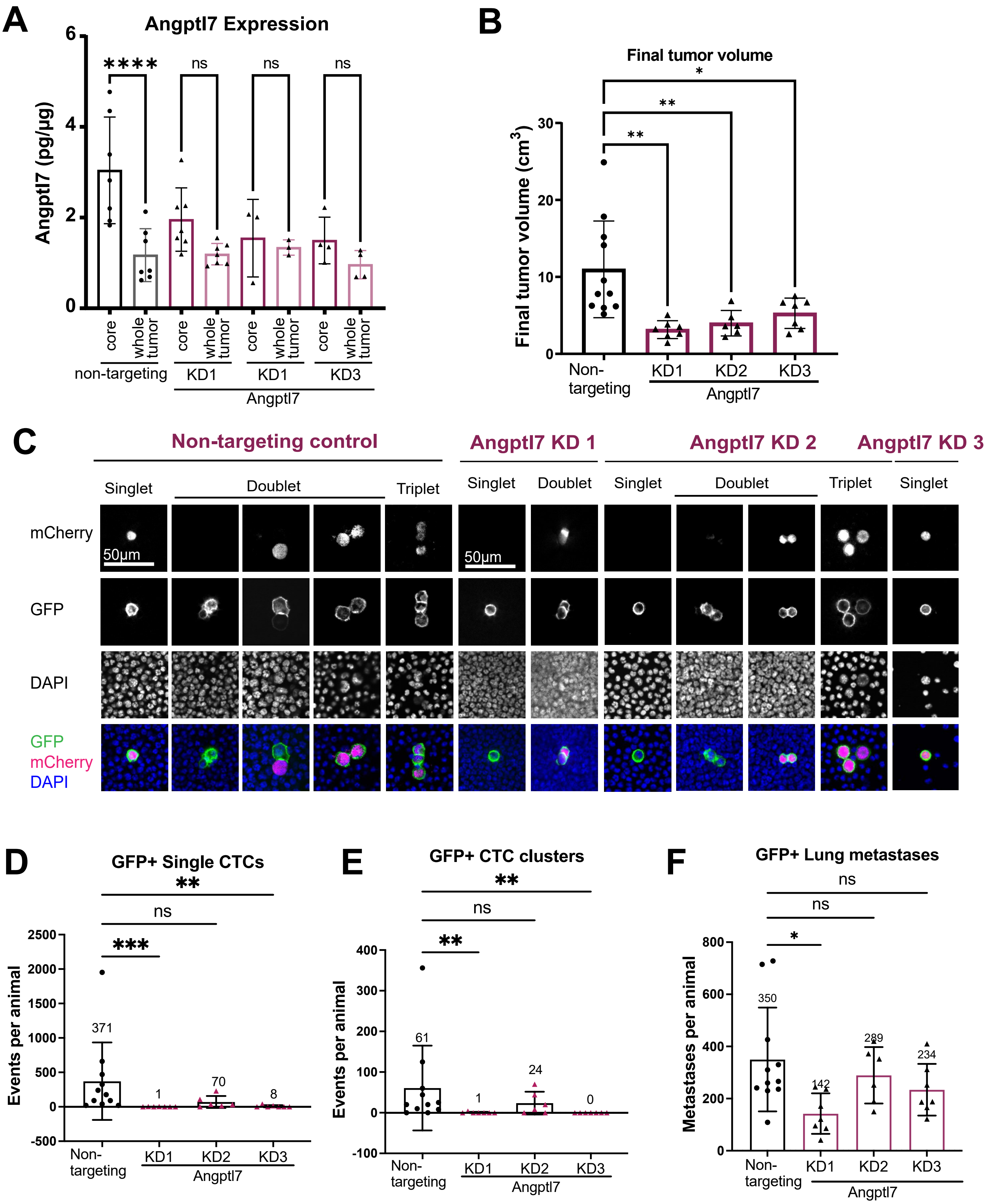
A
Angptl7 Expression
Angptl7 (pg/µg)
****
ns
ns
ns
core
whole tumor
non-targeting
KD1
KD1
KD3
Angptl7
B
Final tumor volume
Final tumor volume (cm3)
*
**
**
Non-targeting
KD1
KD2
KD3
Angptl7
C
Non-targeting control
Angptl7 KD 1
Angptl7 KD 2
Angptl7 KD 3
Singlet
Doublet
Triplet
mCherry
GFP
DAPI
50µm
D
GFP+ Single CTCs
Events per animal
**
ns
***
371
1
70
8
E
GFP+ CTC clusters
61
1
24
0
F
GFP+ Lung metastases
Metastases per animal
*
350
142
289
234

Figure S7

A

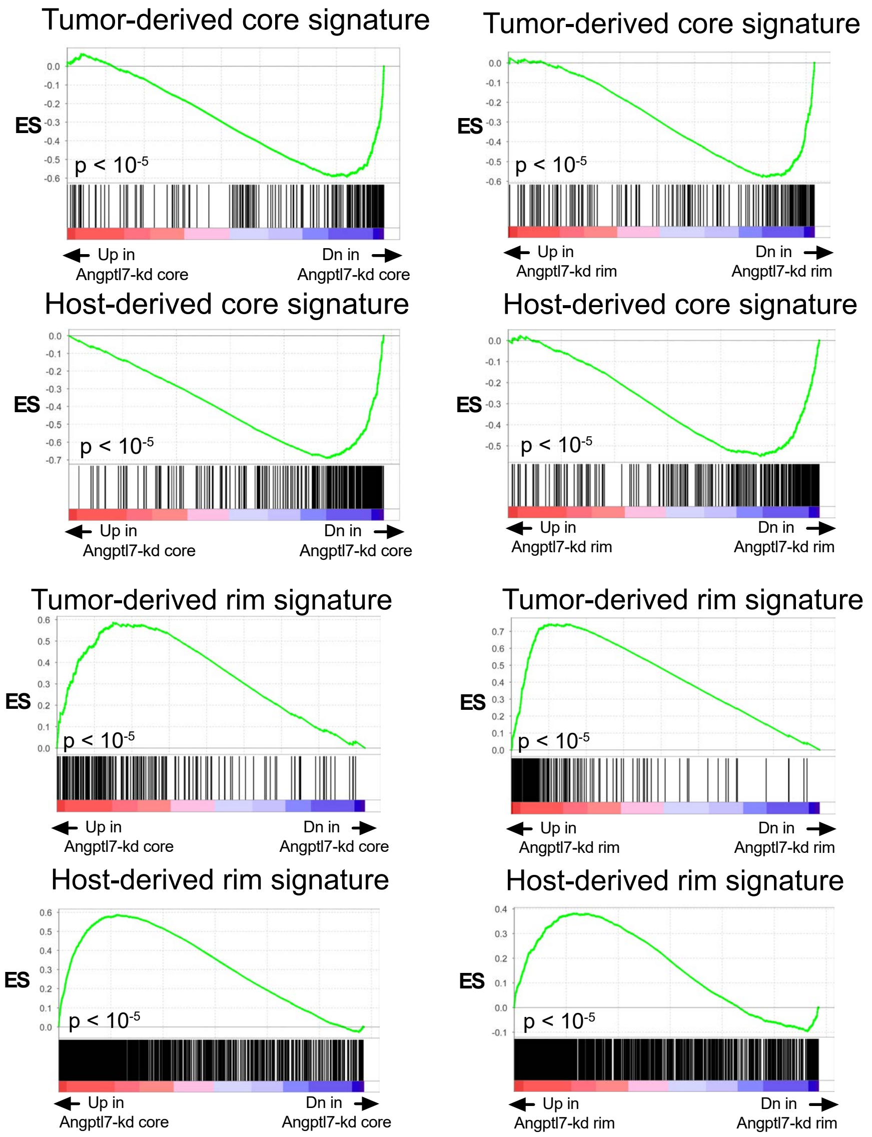

B

	Up in Angptl7 KD	Down in Angptl7-kd
Tumor-derived	239 (23%)	788 (77%)
Host-derived	739 (35%)	1361 (65%)

C

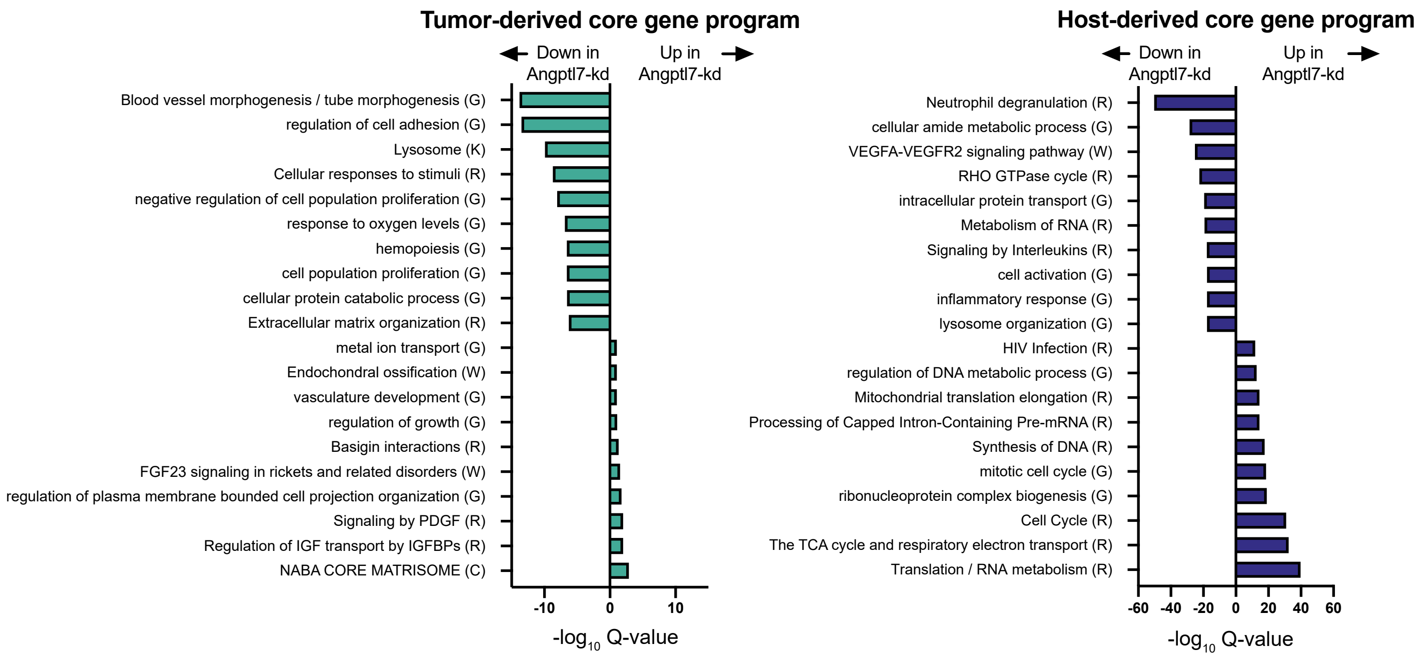

Figure S8

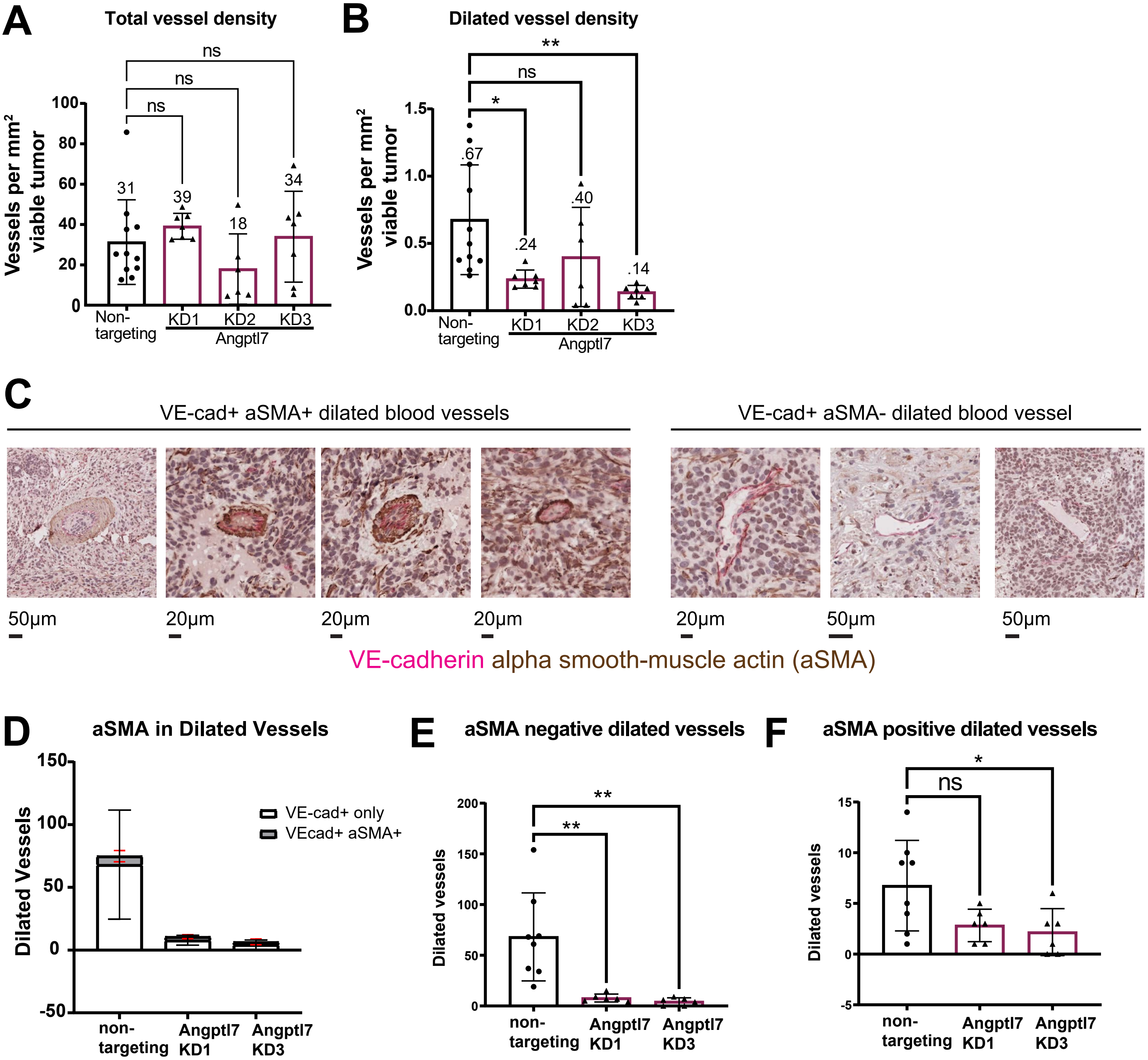
A
Total vessel density
Vessels per mm^2 viable tumor
ns
ns
ns
31
39
18
34
100
80
60
40
20
0
Non-targeting
KD1
KD2
KD3
Angptl7
B
Dilated vessel density
Vessels per mm^2 viable tumor
**
ns
*
.67
.24
.40
.14
1.5
1.0
0.5
0.0
Non-targeting
KD1
KD2
KD3
Angptl7
C
VE-cad+ aSMA+ dilated blood vessels
VE-cad+ aSMA- dilated blood vessel
50µm
20µm
20µm
20µm
20µm
50µm
50µm
VE-cadherin alpha smooth-muscle actin (aSMA)
D
aSMA in Dilated Vessels
Dilated Vessels
150
100
50
0
-50
VE-cad+ only
VEcad+ aSMA+
non-targeting
Angptl7 KD1
Angptl7 KD3
E
aSMA negative dilated vessels
Dilated vessels
**
**
200
150
100
50
0
non-targeting
Angptl7 KD1
Angptl7 KD3
F
aSMA positive dilated vessels
Dilated vessels
*
ns
15
10
5
0
-5
non-targeting
Angptl7 KD1
Angptl7 KD3

Figure S9

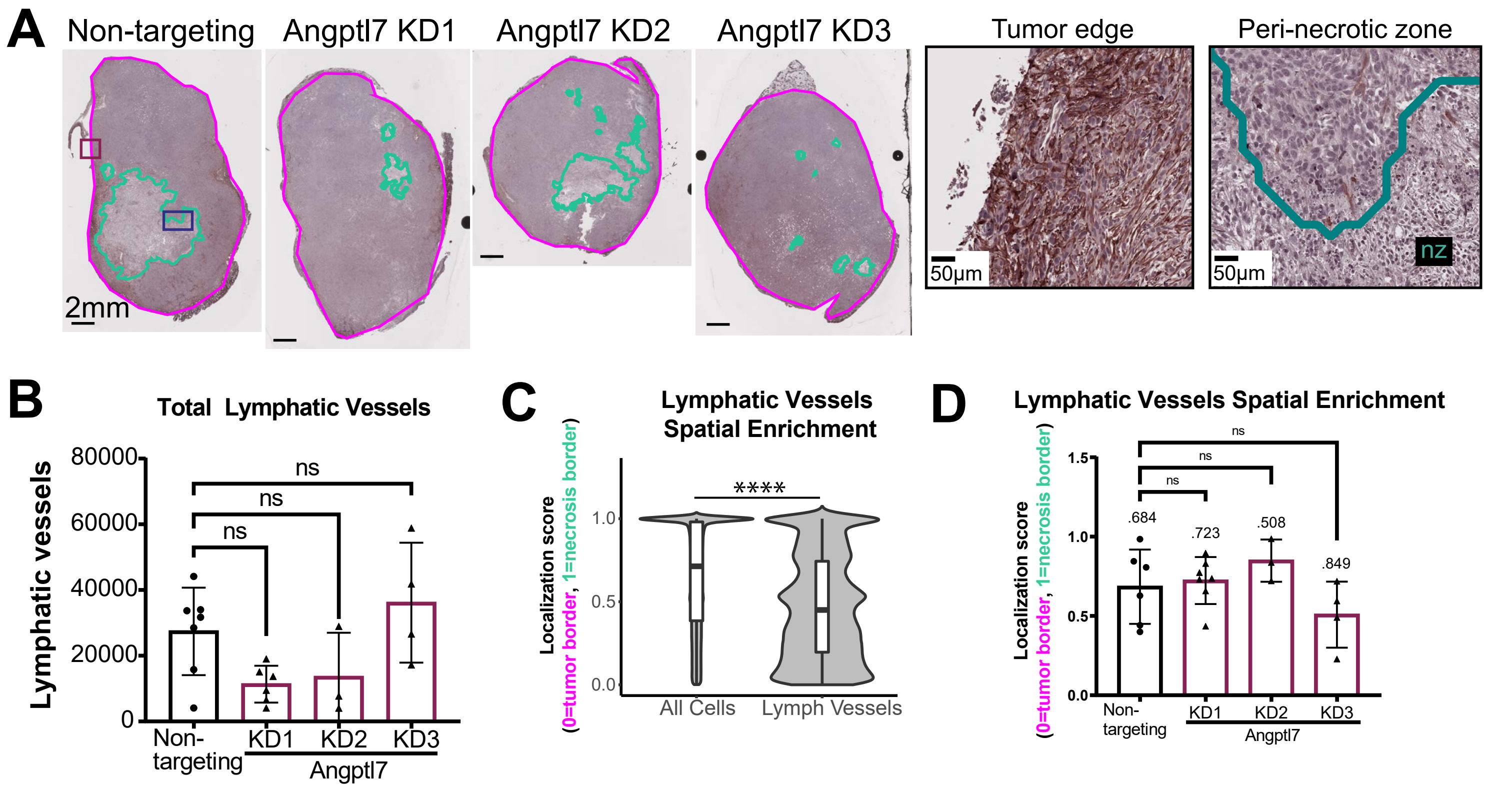
A
Non-targeting
Angptl7 KD1
Angptl7 KD2
Angptl7 KD3
Tumor edge
Peri-necrotic zone
2mm
50µm
50µm
nz
B
Total Lymphatic Vessels
Lymphatic vessels
80000
60000
40000
20000
0
ns
ns
ns
Non-targeting
KD1
KD2
KD3
Angptl7
C
Lymphatic Vessels Spatial Enrichment
Localization score (0=tumor border, 1=necrosis border)
1.0
0.5
0.0
****
All Cells
Lymph Vessels
D
Lymphatic Vessels Spatial Enrichment
Localization score (0=tumor border, 1=necrosis border)
1.5
1.0
0.5
0.0
ns
ns
ns
.684
.723
.508
.849
Non-targeting
KD1
KD2
KD3
Angptl7

Figure S10

CTC dynamics and necrosis in patient with metastatic breast cancer

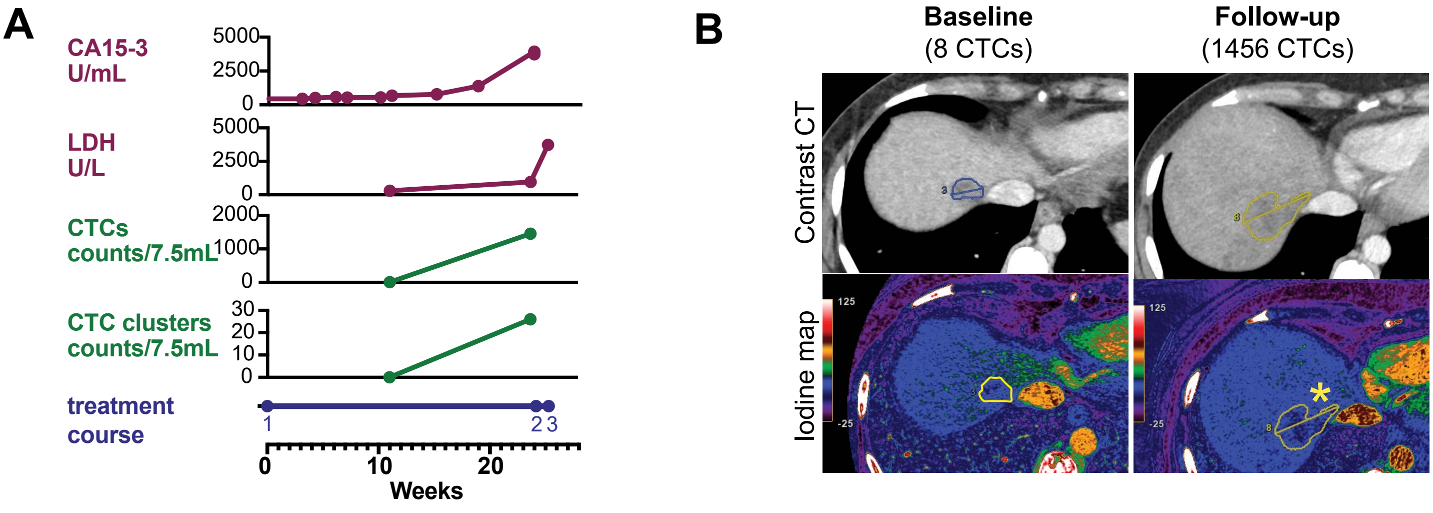

LDH is associated with rising CTC counts and high CTC burden

C

Plasma protein	Fold change
CRP	3.6
HSPA1B	3.4
HSP90AA1	3.2
PSMA3	2.8
LDHA	2.7
ALDOA	2.6
PSMA6	2.6
GPI	2.5
PSMB7	2.5
IGFBP1	2.5
YWHAG	2.4
TALDO1	2.3
ENO1	2.2
EZR	2.1
PSMB1	2.1
GSS	2.0
PSMA7	2.0

necrosis associated

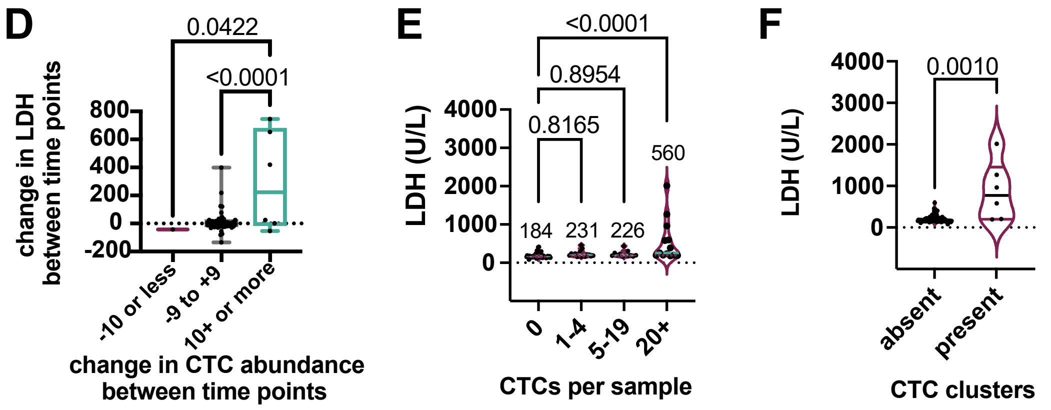

Supplementary Figure Legend

Supplemental Figure S1: Orthotopic transplantation into rats produce 3x larger tumors, 10x more CTCs, and 4x more lung metastases than into mice.

S1A) Experimental schema. 4T1 mouse mammary tumor cells labeled with membrane GFP (4T1-GFP) were orthotopically transplanted into a single #4 mammary fat pad of SRG rats (n=6) or NSG mice (n=24). Animals were sacrificed at 24 days post-transplantation.

S1B-C) Final tumor weight and estimated final tumor volume per animal.

S1D) Blood volume collected per animal.

S1E) Representative micrographs of single CTCs and CTCs clusters from 4T1-GFP transplanted SRG rats. GFP denoted in green. DAPI in blue.

S1F-G) Single CTC and CTC cluster abundance per animal. Individual blood samples from 8 mice were pooled into one tube for a total of n=3 samples. Events per individual animal are reported.

S1H) Percentage of CTC events that were single CTCs or CTC clusters. Mean ± SD.

S1I) Representative stereomicroscope images of lung metastases from NSG mice and SRG rats.

S1J) The number of lung metastases determined by stereomicroscopy in transplanted SRG rats and NSG mice.

Mean values shown on graphs. All P-values determined by Welch’s t-test.

Supplemental Figure S2: Additional morphometric parameters and their correlation with low to high CTC transition

S2A-B) Estimated tumor volume and final tumor weight determined from animals collected as in Fig. 1A. Mean ± SD.

S2C-D) Single CTC and CTC clusters per animal plotted on log scale. Mean ± SD.

S2E) Representative H&E images of the invasive tumor border.

S2F) Representative images of TTC stained tumors. Red areas are viable tissue while white areas are necrotic tissue. Unstained control is provided. Yellow borders indicate necrotic region.

S2G-H) Total area of necrosis and viable area determined from the TTC assay. n=4 animals per time point; 2 to 3 slices per tumor. Mean ± SD.

S2I) Lung metastases per animal. Mean ± SD.

S2J-K) Correlation between lung metastases and necrotic area (J) and lung metastases and tumor size (K). Pearson R. Linear trend lines shown with 95% confidence intervals.

P-values for A-D and I determined by one-way ANOVA. P-Values for G-H determined by Welch's t-test.

Supplemental Figure S3: Additional information on characteristics of blood vessels.

S3A) Dilated blood vessel spatial localization. Distance of VE-cadherin-positive dilated vessels to necrosis or tumor border was measured then normalized to a spatial proximity score of 0 to 1, where 0 means a spot is on the tumor border, and 1 means a spot is in a necrotic region. Spatial localization of all cells were plotted as a control. P-value determined by Kruskal-wallis test.

S3B) Dilated VE-cadherin vessel density per mm^2 viable tumor area determined per animal. Mean ± SD.

S3C) Total VE-cadherin abundance determined per animal. Mean ± SD.

S3D) Total VE-cadherin vessel density per mm^2 viable tumor area determined per animal. Mean ± SD.

S3E-H) Correlation between lung metastasis or CTC events with dilated vessels (E) and total vessels (F). Correlation between CTC events and dilated vessels (G) and total vessels (H). Pearson R. Linear trend lines shown with 95% confidence intervals.

S3I) Representative immunofluorescence images of dilated and non-dilated blood vessels in 4T1 tumors perfused with lectin-594 and stained for VE-cadherin.

S3J) Percent lectin-positive VE-cadherin-positive blood vessels in 4T1 transplant tumors. Mean ± SD.

P-values for B-D determined by one-way ANOVA; J by paired t-test.

Supplemental Figure S4: Additional information on tumor core transcriptional profiling.

S4A) Representative images of necrotic cores of primary tumors used for bulk RNA-seq.

S4B) Percent of transcripts which mapped to the mouse or rat genome. 4T1 cell pellets served as control for mouse-only samples, and rat mammary tissue was used as rat-only samples. Average percentage and standard deviations are shown in the table.

S4C) Top tumor or host-derived gene sets enriched in the tumor core and rim, based on Metascape analysis. Q-value (FDR- False discovery rate) ≤ 0.01 was used as the cut-off for the

gene list. R= reactome, W= WikiPathways, G= GO ontology, K= KEGG C= Canonical Pathways.

S4D) Relative RNA abundance of tumor or host-derived Camk1d expression in the tumor core or rim. Camk1d is the #1 most enriched tumor-derived gene in the tumor core. Camk1d is also expressed by the host and is not tumor specific. Average expression and q-value (FDR) from multiple comparisons indicated on the graph.

S4E) Angptl7 expression in 4T1 tumor core, 4T1 tumor rim, and 2D culture 4T1 cells based on qPCR. Mann-Whitney. Average values shown.

S4F) Western blot of necrotic core and non-necrotic rim regions of 4T1 tumors to assess protein-level differential expression of Angptl7. Angptl7 is highly expressed in necrotic region of the tumor but not the non-necrotic region. GAPDH was used as loading control. 4T1 cells in vitro do not express Angptl7 and was used as the negative control. N=3 rat tumors.

S4G) Quantification of protein-level expression of Angptl7 from S3F. P-value determined by paired t-test.

S4H) Angptl7 spatial localization. Distance of Angptl7+ spots to either necrosis or tumor border was measured then normalized to a spatial proximity score of 0 to 1, where 0 means a spot is on the tumor border, and 1 means a spot is in a necrotic region. Spatial localization of all cells are plotted as a control. P-value determined by Kruskal-wallis test.

Supplemental Figure S5: *Angptl7* expression increase with day post-transplantation, correlates with necrotic area, lung metastases, and dilated vessels.

S5A) *Angptl7* detections from day 13 to day 27 post-transplantation based on RNA ISH. P-values determined by one-way ANOVA.

S5B-E) Correlation between Angptl7 detections per tumor and necrotic area, lung metastases, dilated blood vessels, or CTC events. Pearson R. Linear trend lines shown with 95% confidence intervals.

Supplemental Figure S6: Additional information on in vivo effects of Angptl7 suppression.

S6A) Quantification of ELISA of necrotic core and whole 4T1 tumors for Angptl7. Angptl7 is highly expressed in necrotic region of the tumor. Non-targeting control (n=7), KD1 (n=7), KD2 (n=3), and KD3 (n=4). Paired t-test.

S6B) Estimated final tumor volume of non-targeting and Angptl7 KD tumors.

S6C) Representative images of single CTCs and CTC clusters from Angptl7 knockdown or non-targeting control transplantation into SRG rats. Cells are mCherry-positive if they express shRNAs. 4T1 cells are labeled by membrane GFP. DAPI marks nuclei.

S6D-E) GFP-positive single CTC and CTC cluster abundance in Angptl7 knockdown and non-targeting control.

S6F) GFP+ lung metastases.

All graphs reported as mean ± SD and p-values determined by one-way ANOVA.

Supplemental Figure S7: Additional information on in vivo effect of Angptl7 suppression on gene expression.

S7A) Gene sets were generated composed of tumor-derived and host-derived core and rim genes with fold change ≥ 2 and FDR ≤ 0.001. The relative enrichment of these 4 gene sets was determined for tumor core or rim from Angptl7 knockdown and non-targeting control.

S7B) Genes within tumor-derived and host-derived necrotic core gene sets with FDR ≤ 0.01 were stratified by t-statistic and divided into genes up or down in Angptl7-kd conditions.

S7C) Metascape analysis of tumor and host-derived tumor core gene program stratified by their change in expression with Angptl7 suppression. Gene set enrichment reported as log Q-value.

Supplemental Figure S8: Additional information on in vivo effect of Angptl7 suppression on blood vessels.

S8A) Total VE-cad+ blood vessel density per mm^2 viable tumor area per animal for Angptl7 KD and non-targeting control tumors.

S8B) Dilated VE-cad+ blood vessel density per mm^2 viable tumor area per animal for Angptl7 KD and non-targeting control tumors.

S8C) Representative imaged of alpha smooth actin immunohistochemistry. VE-cadherin in vector red (pink), alpha smooth actin in NOVA Red (brown), counterstained with hematoxylin (purple).

S8D) Alpha smooth actin-positive (aSMA) coverage on VE-cadherin positive dilated vessels in non-targeting control and Angptl7 KD tumors.

S8E) Number of VE-cadherin positive blood vessels with no aSMA coverage in non-targeting control and Angptl7 KD tumors.

S8F) Number of VE-cadherin positive blood vessels with aSMA coverage in non-targeting control and Angptl7 KD tumors.

All graphs shown as mean ± SD. P-values determined by one-way ANOVA.

Supplemental Figure S9: In vivo effect of Angptl7 suppression on lymphatic vessels.

S9A) Representative images podoplanin+ lymphatic vessels in Angptl7 knockdown and non-targeting control tumors. Immunohistochemistry for podoplanin (DAB) (brown) counterstained with hematoxylin. Turquoise: necrotic region, pink: tumor border. Navy border inset is an example of the tumor edge. Magenta border inset is an example of a peri-necrotic region.

S9B) Number of podoplanin+ lymphatic vessels in the Angptl7 knockdown and non-targeting control tumors. Mean ±SD. One-way ANOVA.

S9C) Spatial enrichment of non-targeting control tumors comparing all cells (control distribution) with all podoplanin+ lymphatic vessels. P-value determined by Kruskal-wallis test.

S9D) Spatial enrichment of podoplanin+ lymphatic vessels in Angptl7 KD and non-targeting control tumors. Mean ±SD. One-way ANOVA.

Supplemental Figure S10: Supplementary information on correlation of CTC and necrosis in breast cancer patients with metastatic disease.

S10A) Clinical vignette of patient with metastatic breast cancer and acute CTC elevation.This was a patient with de novo stage IV ER+PR+HER2- metastatic breast cancer involving the right breast, lymph node, bones, liver and lung. Despite initial response to endocrine therapy in combination with the CDK4/6 inhibitor palbociclib, the patient progressed and was switched to capecitabine monotherapy. Over a 30-week period, the patient demonstrated increasing blood levels of tumor marker CA15-3, indicating progressive resistance to therapy. At week 4, an initial CTC determination yielded a CTC count of 8 per 7.5 mL and no CTC clusters. At week 24, a repeat CTC determination showed a CTC count of 1456 per 7.5 mL and 26 CTC clusters per 7.5 mL. Strikingly, lactate dehydrogenase (LDH), a marker of tissue necrosis, also increased over the same period: 309 at week 4 to 963 at week 24 and 3727 U/L at week 26, during inpatient admission for workup of abdominal pain, liver injury, and tumor lysis. Treatment course: (1) capecitabine, (2) in patient admission for liver injury and tumor lysis, (3) death. CA15.3, cancer antigen 15-3; LDH, serum lactate dehydrogenase.

S10B) Dual energy scanning contrast tomography. Baseline scan obtained 1 month prior to first draw (CTC=8 per 7.5 mL). Follow-up scan obtained 1 week after second draw (CTC=1456 per 7.5 mL) during in hospital admission. Upper panels: contrast enhanced CT. Lower panels: iodine distribution. The right lobe liver metastasis (yellow outline) measured 3.1 cm at baseline with a maximum iodine concentration of 38 µg/cm3 and an average of 19.2 µg/cm3 which grew to 4.7 cm on follow up 3 months later and the maximum iodine concentration decreased to 32

μg/cm3 and the average to 14.2 μg/cm3. Lower iodine concentration is indicative of decreased perfusion and increased necrosis.

S10C) Tandem-mass tag mass spectrometry of late versus early time points for low-to-high CTC transitions (n=3 patient sample pairs). Volcano plot shows the plasma proteins enriched in high CTC samples with P-values less than 0.1. Metascape analysis of proteins reported by -log Q-value. Listed are proteins with 2-or-more fold-enrichment in high CTC samples. Necrosis-associated proteins highlighted in blue.

S10D) Change in LDH between time points according to change in CTC abundance between time points. P-values determined by one-way ANOVA comparing against 10+ or more CTCs.

S10E) Serum LDH in relation to CTCs per sample. N=39 patients, 93 blood samples. P-values determined by one-way ANOVA comparing against 0 CTCs.

S10F) Serum LDH in relation to presence or absence of CTC clusters. N=39 patients, 93 blood samples. P-values determined by Mann-Whitney test.

Experimental Model and Subject Details

Animal models

All mice were maintained under specific-pathogen-free conditions, and experiments conformed to the guidelines as approved by the Institutional Animal Care and Use Committee of Fred Hutchinson Cancer Research Center (FHCC). The SRG OncoRats, **S**CID rats on the Sprague-Dawley background that harbors a double knockout for the **R**ag2 and Il2r**g**amma genes (SRG), were purchased from Hera Biolabs. Similar to NSG mice, SRG rats are a double knockout for the Rag2 and Il2rgamma genes (SD-Rag2tm2hera Il2rgtm1hera) and lack B-cells, T-cells, and NK-cells. For mouse experiments, NSG mice (NOD.Cg-Prkdcscid Il2rgtm1Wjl/SzJ) were used. 8-13 week old female mice and rats were used for all experiments.

Human breast cancer patient samples

Blood from patients were obtained from consenting patients under a Fred Hutch IRB approved study (FH8649) for longitudinal monitoring of circulating tumor cells in metastatic breast cancer patients. CTCs and CTC-clusters were enumerated in these fluid samples using a RareCyte assay[1]. For details on the receptor status and pathology of each deidentified human sample used, see **Supplementary Table 1.**

Cell lines

293FT (ThermoFisher Scientific R70007) cell line was grown at 37°C, 5% CO2 in DMEM high glucose, GlutaMAX supplement, pyruvate (GIBCO 10569-010) supplemented with 10% fetal bovine serum (Sigma-Aldrich F0926-500ML) and 1% penicillin/streptomycin (Sigma-Aldrich P4333). 4T1 (ATCC CRL-2539™) and HCC70 (ATCC CRL2315) cell line was grown at 37°C, 5% CO2 in RPMI, GlutaMAX supplement (GIBCO 61870-127) supplemented with 10% fetal bovine serum (Sigma-Aldrich F0926-500ML) and 1% penicillin/streptomycin (Sigma-Aldrich P4333). For non-adherent culture, cell lines were cultured in complete media + 2% (v/v) Matrigel. All cell lines used were from human females. 4T1 Cells were purchased from American Type Culture Collection (ATCC). 4T1-cyto-eGFP cells were a gift from the Cyrus Ghajar at Fred Hutchinson Cancer Center. 4T1-AcGFP1-mem-9 cells were generated by transducing 4T1 cells from ATCC with the rLV.EF1.AcGFP1-Mem-9 lentiviral particle (MOI=5) (Takara Bio). 4T1-AcGFP1-mem-9 cells were selected with 10ug/ml puromycin for at least 2 weeks.

Methods:

2D Cell Culture:

4T1 cells were cultured 2D on adherent plates in RPMI +10% FBS + 1% Penicillin-Streptomycin solution. 4T1-AcGFP1-mem-9 were cultured with RPMI complete + 10ug/ml puromycin. Angptl7 knockdowns and non-targeting control 4T1 cells were cultured with RPMI complete + 10ug/ml puromycin + 10ug/ml blasticidin.

3D Cell Culture:

2D cultured cells were trypsinized with 0.25% trypsin, then quenched with complete RPMI. Cell suspension was centrifuged at 400g for 5mins, then resuspended in Accumax. Accumax suspension was place in 37°C water bath for 30mins, pipetting up

and down every 10 minutes. After centrifuging for 5min at 400g, pellet was resuspended in complete RPMI + 2% Corning® Matrigel® Growth Factor Reduced (GFR) Basement Membrane Matrix (354230) (Corning). Cells were plated in non-adherent 6 well plates at 150,000 cells/mL with 4mL media per well.

Generating knockdown 4T1 cell lines:
4T1-AcGFP1-mem-9 cells were transduced with lentiviral particles generated with shERWOOD UltramiR Lentiviral Inducible shRNA (TransOMIC) for Angptl7 and selected with 10ug/ml puromycin + 10ug/ml blasticidin for at least 2 weeks.

SRG rats:
The SRG OncoRats, **S**CID rats on the Sprague-Dawley background that harbors a double knockout for the **R**ag2 and Il2r**g**amma genes, were purchased from Hera Biolabs. SRG rats are a double knockout for the Rag2 and Il2rgamma genes (SD-Rag2tm2hera Il2rgtm1hera) and lack B-cells, T-cells, and NK-cells.

Orthotopic transplantation into mammary fat pad of rats and mice:
Cells to be transplanted were cultured 3D for 2 passages before being transplanted. After the second passage, cells were plated in non-adherent 6 well plates at 150k cells/mL with 4mL media per well for the cells to aggregate over 24 hours before being transplanted. Spheroids formed this way were resuspended in 1:1 Matrigel:DMEM/F12 mix, kept on ice. 600,000 cells in 20µl Matrigel DMEM/F12 mix were transplanted into the right T4 mammary fat pad of rats or mice. Estimated tumor volume was calculated based on caliper measurements with the formula: $V = (W^2 \times L)/2$.

Blood collection from rats and mice:
For rats, peripheral blood was collected terminally though the left ventricle of the heart, into CellSearch CellSave tubes (Menarini-Silicon Biosystems) with heparin-coated syringe attached to an 18g needle. Rats were put under deep anesthesia with isoflurane to do this, and rats were euthanized immediately after blood collection. Blood tube was inverted at least 10 times immediately after blood collection, and blood was processed within 4 hours of collection. For mouse blood, peripheral blood of 8 mice were collected into one CellSave tube using a new heparin coated syringe for every mouse.

Density separation of buffy coat (nucleated cells) from blood:
Peripheral blood was diluted 1:1 with D-PBS then layered on top of 8mL of Ficoll (GE Ficoll Paque Plus GE17-1440-02 for human blood and Ficoll Paque Premium GE17-5442-02 for mouse and rat blood) in a 2.5% BSA-coated 50mL conical tube. Tube with Ficoll and diluted blood were centrifuged at 400g for 35mins with 0 acceleration and deceleration. The cloudy, white layer ("buffy coat") between the clear Ficoll layer and the top plasma layer and the plasma layer were collected into a separate BSA-coated tube. Buffy coat and plasma layer mix were centrifuged at 4°C at 3500g for 30mins to pellet nucleated cells, then resuspended with D-PBS. To visualize Buffy Coat containing CTCs, cell suspension was spun onto slides using a Cytospin (800g for 5mins). After drying slides completely, slides were fixed with 4% PFA for 5 mins, washed with D-PBS 5mins 3 times. Slides were dried completely again before being stored in -80°C. For

plasma collection, plasma layer was collected from the plasma layer from the Ficoll density separation or from a separate blood tube in an AccuCyte Blood collection tube processed by Rarecyte with a 3000g 25 minutes spin at 25°C.

Immunofluorescence staining:
Slides were thawed at room temp for 5 minutes. For OCT tissue sections, slides were washed 5 mins 3x to wash OCT off. Slides were block for 1 hour with blocking solution (2.5% BSA, 5% normal goat serum or normal donkey serum, 0.3% triton in D-PBS). Primary antibody in blocking solution was placed on slides and incubated at room temp for 2 hours. We then washed the slides 5mins 3x with D-PBS before placing the secondary antibody in 5% normal goat serum or normal donkey serum in D-PBS on the slide to incubate for 1 hour. Slide was washed again 5mins 3x with D-PBS. Excess moisture was removed from the slide, and slide was mounted with 30-50µl Prolong Diamond Antifade Mountant and glass coverslip. Slide was cured overnight room temp, then stored at 4°C.

Bulk RNA-seq:
4T1 cells were transplanted into the right #4 mammary fat pad of SRG rats. At day 27 post-transplantation, primary tumors were harvested. Tumor was cut in half to expose the cross section. The inner, necrotic region and the outer, non-necrotic region were separated and snap frozen for RNA-seq and sent to Genewiz-Azenta for RNA extraction, library prep, and sequencing. Qiagen RNeasy kit (Qiagen, Inc 74104) was used for in-house RNA extraction before samples were sent to Genewiz.

Azenta Genewiz RNA-seq:
Sample QC, library preparations, sequencing reactions, and initial bioinformatic analysis were conducted at GENEWIZ, LLC./Azenta US, Inc (South Plainfield, NJ, USA) as follows:

Sample QC:
Total RNA samples were quantified using Qubit 2.0 Fluorometer (Life Technologies, Carlsbad, CA, USA) and RNA integrity was checked with 4200 TapeStation (Agilent Technologies, Palo Alto, CA, USA).

Library Preparation and Sequencing:
Samples were initially treated with TURBO DNase (Thermo Fisher Scientific, Waltham, MA, USA) to remove DNA contaminants. The next steps included performing rRNA depletion using QIAseq® FastSelect™−rRNA HMR kit (Qiagen, Germantown, MD, USA), which was conducted following the manufacturer's protocol. RNA sequencing libraries were constructed with the NEBNext Ultra II RNA Library Preparation Kit for Illumina by following the manufacturer's recommendations. Briefly, enriched RNAs are fragmented for 15 minutes at 94°C. First strand and second strand cDNA are subsequently synthesized. cDNA fragments are end repaired and adenylated at 3'ends, and universal adapters are ligated to cDNA fragments, followed by index addition and library enrichment with limited cycle PCR. Sequencing libraries were validated using the Agilent Tapestation 4200 (Agilent Technologies, Palo Alto, CA, USA), and quantified

using Qubit 2.0 Fluorometer (ThermoFisher Scientific, Waltham, MA, USA) as well as by quantitative PCR (KAPA Biosystems, Wilmington, MA, USA). The sequencing libraries were multiplexed and clustered on one lane of a flowcell. After clustering, the flowcell was loaded on the Illumina HiSeq 4000 instrument according to manufacturer's instructions. The samples were sequenced using a 2x150 Pair-End (PE) configuration.

Initial Bioinformatics analysis:
Image analysis and base calling were conducted by the HiSeq Control Software (HCS). Raw sequence data (.bcl files) generated from Illumina HiSeq was converted into FASTQ files and de-multiplexed using Illumina's bcl2fastq 2.17 software. One mismatch was allowed for index sequence identification.

RNA-seq computational deconvolution of mouse and rat genomes:
RNA-seq reads in xenograft samples were deconvolved for identification of the species of origin following a method similar to that described by Wingrove et al.[2]. A catenated genome was formed by catenating the Rat (Rnor_6.0) and mouse (GRCmm28) genomes and gene annotations. Prior to alignment, reads were trimmed using Trim Galore and read quality was confirmed using fastQC. Trimmed reads were identified as originating from either rat or mouse by aligning reads against the catenated genome using STAR. Paired reads with both mates aligning uniquely to either the rat or mouse genome were separated by species and counted to gene annotations using featureCounts[3] for downstream differential expression analysis.
Within-species normalization of mapped reads and differential expression analysis were performed using the limma (version 3.50.0)[4] and edgeR (version 3.36.0)[5] packages in R following the procedure described in detail by Law et al.[6]. Prior to differential expression analysis, very low abundance genes were filtered using the filterByExpr function in edgeR using default parameters. Effective library sizes were normalized across conditions using the trimmed mean of M-values (TMM) normalization procedure. Differential expression analysis was performed on the filtered and normalized counts using the limma-voom linear modeling pipeline[7]. For experiments in which samples were paired by animal, animal ID was treated as an additional random effect in the linear model. Empirical Bayes smoothing was applied on both the linear model and fitted contrast coefficients using edgeR. False-discovery rates for differential expression at the gene-level were calculated using the Benjamini-Hochberg correction procedure with the toptable function in limma. Normalized transcript abundances are reported as the TMM-normalized counts-per-million obtained from the voom function in limma.
Gene set enrichment analysis was performed using GSEA software (Broad Institute, v 4.2.3)[8]. Genes were ranked by log-fold change and collapsed to human orthologs using the MDSig ensembl gene id orthologs chip platform (v 7.3). GSEA was performed using a pre-ranked analysis weighted by log-fold change against the C5 gene ontology collection (v 7.5). False-discovery rates were determined using 100,000 permutations.
For differential expression analysis between species (tumor and host), a modified normalization scheme was applied to account for differences in gene lengths and annotation depth between species, in addition to the standard normalization by effective library size. Our method resembles that described by Oziolor, et al[9]. First, using reads

that mapped uniquely to one species of the catenated genome, an intra-species TMM normalization was performed separately for host- and tumor-mapped reads. Length-corrected transcript abundances were calculated as reads per kilobase million (RPKM) in edgeR, and then converted to transcripts per million (TPM). Homolog pairs were obtained by querying the Ensembl database for linked rat (rattus norvegicus Ensembl release 100) and mouse (mus musculus Ensembl release 100) datasets using the getLDS function in biomaRt (v 2.50.3). Next, both gene sets were filtered to only those with rat/mouse homolog pairs, and the rat genes were mapped onto their corresponding mouse homologs. TPM abundances originating from both species were combined into a single data set, treating species of origin as a sample identifier, for all further analysis. To correct for differences in genome annotation depth between species, TPM values were normalized using the TMM method in edgeR. Homolog-wise differential expression analysis was performed between species using TMM-normalized, homolog-mapped TPM values as input into the limma-voom analysis pipeline (as above) in place of library-normalized counts. Prior to identifying top genes that were differentially expressed in the mouse tumor over the rat host, homolog pairs that exhibited zero read counts in the rat homolog for all of the samples were discarded, since these may reflect a limitation of the homolog mapping. Then, genes from mouse-core-mouse-rim were cross referenced with mouse-core-rat-core. Genes that were mouse-core vs. mouse-rim Log Fold Change ≥ 1 and FDR ≤ 0.01 and with detectable rat expression are shown in Fig 4C.

Real Time qPCR:
RNA from tumors and cell pellets were extracted using Qiagen RNeasy kit. RNA was reverse transcribed into cDNA using Superscript™ III First-Strand Synthesis System (Thermo Fisher Scientific Catalog number: 18080051) with equivalent amounts of RNA (1µg). cDNAs were mixed with indicated gene-specific primers listed and PowerUp SYBR Green Master Mix (Thermo Fisher Scientific Catalog number: A25741) and qRT-PCR was performed using an Applied Biosystems QuantStudio5 System.

Immunohistochemistry (IHC):
Tumors were sliced into ~5mm sections immediately after harvest, placed into histology cassettes(#15181701A), and fixed with 10% neutral buffered formalin solution (#HT501128) at room temperature for 5 days. Cassetted tumor sections were paraffin processed, embedded, and cut by Experimental Histopathology Core at Fred Hutchinson Cancer Research Center. Paraffin-embedded tumor slides were warmed in 56°C incubator for 20min in slide holder. Tissue was deparaffinized with Histo-Clear solution (#64111-04) twice for 5min each. Rehydration was performed by immersing the slides through the following solutions twice for 3min each: 100% ethanol, 95% ethanol, 70% ethanol, and deionized water. Heat-induced antigen retrieval was completed by incubating slides in pressure cooker at 6psi (~105°C) for 10-15min with Tris-EDTA (10mM Tris Base, 1mM EDTA, 0.05% Tween20, pH9.0) or sodium citrate (10mM sodium citrate, 0.05% Tween20, pH6.0) buffers. Endogenous peroxidase activity was blocked by 3% H_2O_2 in PBS for 20min. Fc receptor was blocked by Fc Receptor Blocker (#NB309) for 30min. Primary antibodies were diluted with blocking solution (2.5% host serum/1.25%BSA in PBS) and incubated on the slides at room temperature for 1hour.

Species specific ImmPRESS™ secondary antibodies [HRP polymer] from Vector Laboratories were applied directly onto the slides and incubated for 30min at room temperature. Substrate was developed using ImmPACT™ NovaRED^{TM} HRP Substrate Kit (#SK-4805). Mayer's Hematoxylin (#26043-05) was used for counterstaining. Dehydration was performed by dipping the slides 10 times through the following solutions twice: 95% Ethanol and 100% Ethanol, followed by immersing the slides in Histo-clear solution twice for 2min each. CytoSeal 60 (#18006) was used to mount slides, then slides were scanned by Ventana DP 200 slide scanner.

Western Blotting:
Snap frozen tumors were milled into powder and 0.1g was dispensed to make lysate. Lysis buffer was prepared by diluting protease (#87785) and phosphatase (#78420) cocktail inhibitors 1:100 in 1X RIPA buffer (#9806S) and added into tumor powder (1mL of lysis buffer/0.1g of powder). The suspension was sonicated for 6 pulses with a probe sonicator (20%duty cycle, 30% amplitude), incubated at 4°C for 1 hour, and centrifuged at 14,000g for 10min to clear out insoluble substance. Protein concentration was quantified using Pierce BCA Assay Kit (#PI23225). Sample loading solution was prepared by combining tumor lysates (volume for 50ug of protein), 3.3uL of 10X Bolt reducing agent (#B0009), 8.25uL of 4X Bolt LDS buffer (#B0007), and MiliQ water to a final volume of 33uL. A Bolt^{TM} 4-12%, Bis-Tris, 12-well, 1.0 mm Mini Protein Gel, (#NW04122BOX) was loaded with 30uL of sample loading solution per well and run at 100V for 20min and then at 170V for 40min using 1X Bolt MES SDS running buffer (#B0002). The protein transfer sandwich was assembled using Immobilon-FL PVDF Membrane (#IPFL07810) and run at 15V for 1 hour with 1X Bolt transfer buffer (#BT00061) containing 10% methanol. The PVDF membrane was blocked with 3% BSA in TBS for 1 hour. Primary antibodies were diluted with 0.2% TBST and incubated overnight at 4°C. Species specific LI-COR 680 and 800 secondary antibodies were diluted 1:10,000 with 0.2% TBST plus 0.01% SDS and incubated for 1 hour at room temperature. The PVDF membrane was imaged with LiCor Odyssey CLx.

Angptl7 ELISA:
Sample loading solution was prepared by combining tumor lysate (volume for 300ug of protein) and sample diluent from the Mouse Angptl7 ELISA Kit ((#MBS612413) to a final volume of 400uL. 100uL of sample loading solution was loaded in each well for three replicates. Each well was incubated with biotinylated Anti-Mouse Angptl7 antibody, followed by Avidin-Biotin-Peroxidase Complex, and then Color Developing Reagent followed by Stop Solution per manufacturer instructions. O.D. absorbance was measured using a microplate reader at 450nm.

Angptl7 RNA ISH:
For in situ hybridization (ISH), formalin-fixed paraffin-embedded tissues were sectioned at 4 microns onto positively-charged slides and baked for 1 hour at 60°C. The slides were then dewaxed and stained on a Leica BOND Rx stainer (Leica, Buffalo Grove, IL) using Leica Bond reagents for dewaxing (Dewax Solution), antigen retrieval with Epitope Retrieval Solution 2 for 15 minutes at 95°C, protease digestion at 40C for 15 minutes and rinsing after each step (Bond Wash Solution). All other steps were

performed at ambient temperature. Staining was performed with RNAscope 2.5 LS Reagent Kit – BROWN (Cat. No. 322100) or RNAscope 2.5 LS Reagent Kit – RED (Cat. No. 322150). Species-specific probes were applied and incubated at 42C for 120 minutes. Probes used were as follows: RNAscope 2.5 LS Positive Control Probe Human PPIB (Cat. No. 313908), RNAscope 2.5 LS Positive Control Probe Mouse PPIB (Cat. No. 313918), RNAscope 2.5 LS Negative Control Probe dapB (Cat. No. 312038), RNAscope 2.5 LS Human Angptl7 (Cat. No. 552818), RNAscope 2.5 LS Mouse Angotl7 (Cat. No. 552528). Chromogenic staining was performed using BOND Polymer Refine Detection (Cat. No. DS9800) or BOND Polymer Refine Red Detection (Cat. No. DS9390). After staining, slides were removed from the stainer, dehydrated and coverslipped. Slides stained with the Brown kit were coverslipped with Epredia Cytoseal XYL (Cat. No. 8312-4) and slides stained wit the Red kit were coverslipped with Axell Crystal Mount (Cat. No. BMDM02).

QuPath quantification of RNA ISH:
RNAscope images were opened in QuPath[10]. Thresholds were created to identify tumor area and necrotic area by eye, verified to be accurate in test images, and applied to demarcate necrotic zones. Angptl7-expressing cells were identified using positive cell detection. The distance to annotation 2d feature was used to determine the distance between cells and the nearest necrotic border and cells and nearest tumor border. This process was automated in QuPath for consistent analysis across images.

Necrosis Measurements:
Tumor necrosis measurements were determined based on Hematoxylin & Eosin (H&E) or 2,3,5-triphenyltetrazolium chloride (TTC) assay. For H&E staining, tumors were sliced into 2-3 ~5mm slices by scalpel, fixed for 5 days in 10% formalin at 4°C, rocking before being sectioned and stained with hematoxylin and eosin. For the TTC assay, tumors were sliced into 2-3 ~5mm slices and stained with 1g/100ml tetrazolium salt in a 7.4pH buffer with 77.4% NaH2PO4 (0.1 M) and 22.6% Na2HPO4 (0.1M) mix, at 37°C for 20 mins, rocking. Tumor slices were then fixed with 10% formalin for 20 mins before being visualized. In the TTC assay, the TTC compound is reduced to a red TPF (1,3,5-triphenylformazan) compound in live tissues due to dehydrogenase activity. White areas therefore indicate necrotic tissue, and red areas are viable regions.

Spatial enrichment localization score:
For each spot x, the spatial enrichment score (SES) was computed as Dt/(Dn+Dt) where Dn equals the distance between spot x and the nearest necrotic border and Dt equals the distance between spot x and the nearest tumor border (Dt). Accordingly, SES(x) varies from 0 to 1: equal to 0 for perfect localization to the tumor border, and 1 for perfect localization to the necrotic border. Distances calculated from QuPath were exported to R and analyzed using a custom script. A non-parametric Kruskal-Wallis test was applied to evaluate statistical significance between different SES distributions.

LC/MS plasma proteomics
<u>Sample Preparation</u>: Each plasma sample was depleted of high-abundance proteins by injecting 80 µL of the sample onto a Michrom Bioresources Paradigm HPLC equipped

with an Agilent human MARS-6 depletion column. Unbound material eluting from the column, as observed on a UV detector, was collected and the protein concentration was determined. The volume corresponding to 100 µg of protein was subjected to the reduction of disulfide bonds by adding TCEP (tris(2-carboxyethyl)phosphine to a final concentration of 5 mM and incubating at room temperature with vortexing for 15 min. Protein alkylation was carried out by adding 2-chloroacetamide to a final concentration of 10 mM and incubating at room temperature for 30 min. The volume of each sample was reduced to approximately 100 µL by vacuum centrifugation and methanol/chloroform precipitation was carried out. Protein pellets were washed with methanol and resuspended in 50 mM HEPES pH 8.7. Proteolytic digestion was initiated with the addition of 1 µg of Lys-C and incubating at room temperature with low vortexing for 2 hours, followed by the addition of 1 µg of trypsin and incubating overnight at 37 °C on an orbital shaker set at 700 rpm.

TMT Labeling: ThermoScientific TMTpro-16plex reagent was brought to room temperature and resuspended in 20 µL of anhydrous acetonitrile and vortexed for 15 min. Labeling was carried out by adding 20 µL of each TMT reagent to its assigned sample and vortexing the samples occasionally at room temperature for 1 hour. A “label check” was performed by combining 2 µL from each labeled sample into one tube, removing the acetonitrile by vacuum centrifugation, desalting the sample on a Harvard Apparatus C18 ultra-micro spin column, and analyzing the desalted TMT-labeled peptides by LC-ESI-MS/MS. After data analysis (see below), the peptide to spectrum match (PSM) results were analyzed and labeling efficiency was determined to be greater than 98%. After this labeling check, hydroxlyamine was added to each labeled sample to a concentration of 0.5% in order to fully quench the labeling reaction. All samples were combined equally by correcting for the total protein abundances measured for each sample in the labeling check analysis. The equalized pool was subjected to vacuum centrifugation to remove acetonitrile and then desalted on a Waters SepPack C18 (3cc, 200 mg) cartridge. The desalted elution was split into equal fractions and taken to dryness. One of the fractions was injected onto a ThermoScientific Vanquish HPLC equipped with an Agilent 2.1 mm x 150 mm C18 Extend column and fractionated into a 96-well plate using basic reverse-phase conditions. The 96 fractions were concatenated into 24 pools (pool 1: fractions 1, 25, 49, 73; pool 2: fractions 2, 26, 50, 74; etc.) that were taken to dryness and each pool was analyzed by LC-ESI-MS/MS.

LC-ESI-MS/MS: The generated basic reverse phase fractions were brought up in 20 µL of 2% acetonitrile in 0.1% formic acid and 5 µL was analyzed by LC/ESI MS/MS with a Thermo Scientific Easy1200 nLC (Thermo Scientific, Waltham, MA) coupled to a tribrid Orbitrap Eclipse with FAIMS (field asymmetric ion mobility spectrometry) mass spectrometer (Thermo Scientific, Waltham, MA). In-line de-salting was accomplished using a reversed-phase trap column (100 µm × 20 mm) packed with Magic C_{18}AQ (5-µm, 200 Å resin; Michrom Bioresources, Auburn, CA) followed by peptide separations on a reversed-phase column (75 µm × 270 mm) packed with ReproSil-Pur C_{18}AQ (3-µm, 120 Å resin; Dr. Maisch, Baden-Würtemburg, Germany) directly mounted on the electrospray ion source. A 120-minute gradient from 4% to 44% B (80% acetonitrile in 0.1% formic acid) at a flow rate of 300 nL/minute was used for chromatographic separations. A spray voltage of 2300 V was applied to the electrospray tip and the

FAIMS source used varied compensation voltages of -40, -60, -80 while the Orbitrap Eclipse instrument was operated in the data-dependent mode. MS survey scans were in the Orbitrap (Normalized AGC target value 300%, resolution 120,000, and max injection time 50 ms) with a 3 sec cycle time and MS/MS spectra acquisition were detected in the Orbitrap (Normalized AGC target value of 250%, resolution 50,000 and max injection time 100 ms) using HCD activation with a normalized collision energy of 35%. Selected ions were dynamically excluded for 60 seconds after a repeat count of 1. Data Analysis**:** Data analysis was performed using Proteome Discoverer 2.4 (Thermo Scientific, San Jose, CA). The data were searched against a Uniprot Human database (UP000005640, Dec 1, 2019) that included common contaminants (cRAPome 2015). Searches were performed with settings for the proteolytic enzyme trypsin. Maximum missed cleavages were set to 2. The precursor ion tolerance was set to 10 ppm and the fragment ion tolerance was set to 0.6 Da. Dynamic peptide modifications included oxidation (+15.995 Da on M). Dynamic modifications on the protein terminus included acetyl (+42.-11 Da on N-terminus), Met-loss (-131.040 Da on M) and Met-loss+Acetyl (-89.030 Da on M) and static modifications TMTpro (+304.207 on N-terminus and K), carbamidomethyl (+57.021 on C). Sequest HT was used for database searching. All search results were run through Percolator for peptide validation and results were filter to a 1% false discovery rate. To delineate differential expression proteins, both pairwise two-sample *t* tests and Wilcoxon rank-sum test were conducted (R package).

Quantification and Statistical Analysis

Bars are presented as mean ± standard deviation. Graphs were created and statistical tests conducted in GraphPad Prism 8. Non-parametric tests were used when data were not normally distributed or when the median was a better representation of the sample than the mean. For animal experiments, each animal was considered a biological replicate. For in vitro experiments, experiments using cell lines on different days were considered biological replicates. P-values denoted as follows: * < 0.05, ** < 0.01, *** < 0.001, **** < 0.0001.

Key Resources Table

REAGENT or RESOURCE	SOURCE	IDENTIFIER
**Antibodies**		
Goat Angiopoietin-like 7 (Mouse)	R&D Systems	AF4960
Rabbit VE-Cadherin	Abcam	ab205336
Mouse α-Smooth Muscle Actin	Sigma	A5228
Syrian hamster Podoplanin	ThermoFisher	14-5381-82

Mouse Pan-Keratin (C11) (Mouse) (Alexa Fluor® 647 Conjugate)	Cell Signaling Technology	4528S
Mouse GAPDH	ProteinTech	60004
Mouse Beta Actin	Abcam	ab6276
**Biological Samples**		
Human breast cancer, blood samples	TARGET CLUSTER Study	See Supplementary Table 5
**Chemicals, Peptides, and Recombinant Proteins**		
RNAscope® 2.5 LS Probe - Mm-Angptl7	ACD Bio-Techne	552828
RNAscope 2.5 LS Probe - Hs-ANGPTL7	ACD Bio-Techne	552818
**Critical Commercial Assays**		
Mouse ANGPTL7 ELISA Kit	MyBioSource	MBS1752641
**Deposited Data**		
RNA sequencing of tumor core vs rim	This paper	Raw RNA-sequencing data have been deposited on the NCBI SRA (BioProjects: PRJNA925873)
RNA sequencing of Angptl7 knockdown vs non-targeting control tumors	This paper	Raw RNA-sequencing data have been deposited on the NCBI SRA (BioProjects: PRJNA925929)
RNA-sequencing of rhANGPTL7 treated HUVECs	This paper	Raw RNA-sequencing data have been deposited on the NCBI SRA (BioProjects: PRJNA928719)
**Experimental Models: Cell Lines**		
Human: 293FT	ThermoFisher Scientific	R70007
Mouse: 4T1	ATCC	CRL-2539

Mouse: 4T1-cyto-GFP	Gift from Cyrus Ghajar	N/A
**Experimental Models: Organisms/Strains**		
Mouse: FVB/NJ	The Jackson Laboratory	001800
Mouse: NOD.Cg-Prkdcscid Il2rgtm1Wjl/SzJ	The Jackson Laboratory	005557
Rat: SRG Oncorat	Hera BioLabs	SRG-01
**Oligonucleotides**		
Mouse *Angptl7* qPCR Primer	Millipore Sigma	KiCqStart M_Angptl7_1
Mouse beta actin qPCR Primer	Millipore Sigma	KiCqStart M_ActB_1
**Recombinant DNA**		
PsPax2	A gift from Didier Trono	Addgene #12260
MD2.G	A gift from Didier Trono	Addgene #12259
shCtrl Non-targeting #4 pZIP-mCMV-mCherry-Blast (non-targeting control)	transOMIC	TLMSU1451
pZIP-mCMV-mCherry-Blasticidin-Mouse-shAngptl7 (Angptl7 knockdown 1)	transOMIC	Ultra-3367559
pZIP-mCMV-mCherry-Blasticidin-Mouse-shAngptl7	transOMIC	Ultra-3367561

(Angptl7 knockdown 2)		
pZIP-mCMV-mCherry-Blasticidin-Mouse-shAngptl7 (Angptl7 knockdown 3)	transOMIC	Ultra-344221
rLV.EF1.AcGFP1-Mem-9	Takara BIO INC.	0019VCT
Recombinant Human Angiopoietin-like 7 Protein	Biotechne R&D	914-AN-025
**Software and algorithms**		
GraphPad Prism 9	Graphpad Software	https://www.graphpad.com/scientific-software/prism/
FIJI v2.0.0-rc-69/1.52p	Schindelin et al., 2012[11]	https://fiji.sc/
R v3.6.1	R Core Team	https://www.r-project.org/
Metascape	Zhou et al., 2019[12]	https://metascape.org/
R v 4.1.1	R Core Team	https://www.r-project.org/
STAR v. 2.7.3	Dobin, et al., 2013[13]	https://github.com/alexdobin/STAR
limma v. 3.5	Ritchie, et al., 2015[14]	https://bioconductor.org/packages/release/bioc/html/limma.html
edgeR v 3.36.0	Robinson, et al. 2010[15]	https://bioconductor.org/packages/release/bioc/html/edgeR.html
biomaRt v. 2.50.3	Durinck, et al. 2009[16]	https://bioconductor.org/packages/release/bioc/html/biomaRt.html
Rsubread v. 2.4.3	Liao, et al., 2019[17]	https://bioconductor.org/packages/release/bioc/html/Rsubread.html
FastQC v. 0.11.9	Babraham Bioinformatics	https://www.bioinformatics.babraham.ac.uk/projects/fastqc/
Trim Galore v. 0.6.5	Babraham Bioinformatics	https://www.bioinformatics.babraham.ac.uk/projects/trim_galore/

SAMtools v. 1.11	Danececk, et al., 2021[18]	https://github.com/samtools/samtools
GSEA v. 4.2.3	Broad Institute	https://www.gsea-msigdb.org/gsea/index.jsp
Necrotic enrichment score algorithm	This paper	Zenodo DOI: 10.5281/zenodo.7574395
RNA-seq deconvolution and differential expression analysis scripts	This paper	The RNA-seq analysis code used to analyze tumor xenograft samples are available on Zenodo: DOI:10.5281/zenodo.7574251 and future versions will be maintained on github: https://github.com/bkrajina/RNA_seq_xenograft_analysis.

SI Appendix References:

1. Kaldjian EP, Ramirez AB, Sun Y, et al. The RareCyte® platform for next-generation analysis of circulating tumor cells. *Cytometry*. 2018;93(12):1220.
2. Wingrove E, Liu ZZ, Patel KD, et al. Transcriptomic Hallmarks of Tumor Plasticity and Stromal Interactions in Brain Metastasis. *Cell Rep*. 2019;27.
3. Liao Y, Smyth GK, Shi W. featureCounts: an efficient general purpose program for assigning sequence reads to genomic features. *Bioinformatics*. 2014;30(7):923-930.
4. Ritchie ME, Phipson B, Wu D, et al. limma powers differential expression analyses for RNA-sequencing and microarray studies. *Nucleic Acids Res*. 2015;43(7):e47-e47.
5. Robinson MD, McCarthy DJ, Smyth GK. edgeR: a Bioconductor package for differential expression analysis of digital gene expression data. *Bioinformatics*. 2010;26(1):139-140.
6. Smyth GK, Ritchie ME, Law CW, et al. RNA-seq analysis is easy as 1-2-3 with limma, Glimma and edgeR. *F1000Research 2018 5:1408*. 2018;5:1408.
7. Law CW, Chen Y, Shi W, Smyth GK. Voom: Precision weights unlock linear model analysis tools for RNA-seq read counts. *Genome Biol*. 2014;15(2):1-17.
8. Subramanian A, Tamayo P, Mootha VK, et al. Gene set enrichment analysis: A knowledge-based approach for interpreting genome-wide expression profiles. *Proc Natl Acad Sci U S A*. 2005;102(43):15545-15550.
9. Oziolor E, Arat S, Martin M. Annotation depth confounds direct comparison of gene expression across species. *BMC Bioinformatics*. 2021;22(1):1-15.
10. Bankhead P, Loughrey MB, Fernández JA, et al. QuPath: Open source software for digital pathology image analysis. *Sci Rep*. 2017;7(1).
11. Schindelin J, Arganda-Carreras I, Frise E, et al. Fiji: an open-source platform for biological-image analysis. *Nature Methods 2012 9:7*. 2012;9(7):676-682.
12. Zhou Y, Zhou B, Pache L, et al. Metascape provides a biologist-oriented resource for the analysis of systems-level datasets. *Nature Communications 2019 10:1*. 2019;10(1):1-10.
13. Dobin A, Davis CA, Schlesinger F, et al. STAR: ultrafast universal RNA-seq aligner. *Bioinformatics*. 2013;29(1):15-21.
14. Ritchie ME, Phipson B, Wu D, Hu Y, Law CW, Shi W, Smyth GK (2015). limma powers differential expression analyses for RNA-sequencing and microarray studies. *Nucleic Acids Research*, 2015; 43(7), e47.
15. Durinck S, Spellman PT, Birney E, Huber W. Mapping Identifiers for the Integration of Genomic Datasets with the R/Bioconductor package biomaRt. *Nat Protoc*. 2009;4(8):1184.
17. Liao Y, Smyth GK, Shi W. The R package Rsubread is easier, faster, cheaper and better for alignment and quantification of RNA sequencing reads. *Nucleic Acids Res*. 2019;47(8):e47.
18. Danecek P, Bonfield JK, Liddle J, et al. Twelve years of SAMtools and BCFtools. *Gigascience*. 2021;10(2):1-4.
